# Effects of copy number variations on brain structure and risk for psychiatric illness: Large‐scale studies from the ENIGMA working groups on CNVs

**DOI:** 10.1002/hbm.25354

**Published:** 2021-02-21

**Authors:** Ida E. Sønderby, Christopher R. K. Ching, Sophia I. Thomopoulos, Dennis van der Meer, Daqiang Sun, Julio E. Villalon‐Reina, Ingrid Agartz, Katrin Amunts, Celso Arango, Nicola J. Armstrong, Rosa Ayesa‐Arriola, Geor Bakker, Anne S. Bassett, Dorret I. Boomsma, Robin Bülow, Nancy J. Butcher, Vince D. Calhoun, Svenja Caspers, Eva W. C. Chow, Sven Cichon, Simone Ciufolini, Michael C. Craig, Benedicto Crespo‐Facorro, Adam C. Cunningham, Anders M. Dale, Paola Dazzan, Greig I. de Zubicaray, Srdjan Djurovic, Joanne L. Doherty, Gary Donohoe, Bogdan Draganski, Courtney A. Durdle, Stefan Ehrlich, Beverly S. Emanuel, Thomas Espeseth, Simon E. Fisher, Tian Ge, David C. Glahn, Hans J. Grabe, Raquel E. Gur, Boris A. Gutman, Jan Haavik, Asta K. Håberg, Laura A. Hansen, Ryota Hashimoto, Derrek P. Hibar, Avram J. Holmes, Jouke‐Jan Hottenga, Hilleke E. Hulshoff Pol, Maria Jalbrzikowski, Emma E. M. Knowles, Leila Kushan, David E. J. Linden, Jingyu Liu, Astri J. Lundervold, Sandra Martin‐Brevet, Kenia Martínez, Karen A. Mather, Samuel R. Mathias, Donna M. McDonald‐McGinn, Allan F. McRae, Sarah E. Medland, Torgeir Moberget, Claudia Modenato, Jennifer Monereo Sánchez, Clara A. Moreau, Thomas W. Mühleisen, Tomas Paus, Zdenka Pausova, Carlos Prieto, Anjanibhargavi Ragothaman, Céline S. Reinbold, Tiago Reis Marques, Gabriela M. Repetto, Alexandre Reymond, David R. Roalf, Borja Rodriguez‐Herreros, James J. Rucker, Perminder S. Sachdev, James E. Schmitt, Peter R. Schofield, Ana I. Silva, Hreinn Stefansson, Dan J. Stein, Christian K. Tamnes, Diana Tordesillas‐Gutiérrez, Magnus O. Ulfarsson, Ariana Vajdi, Dennis van 't Ent, Marianne B. M. van den Bree, Evangelos Vassos, Javier Vázquez‐Bourgon, Fidel Vila‐Rodriguez, G. Bragi Walters, Wei Wen, Lars T. Westlye, Katharina Wittfeld, Elaine H. Zackai, Kári Stefánsson, Sebastien Jacquemont, Paul M. Thompson, Carrie E. Bearden, Ole A. Andreassen, for the ENIGMA‐CNV Working Group, Manon Bernard, Nicholas B. Blackburn, Rune Bøen, Eco de Geus, Sonja M. C. de Zwarte, Marta Di Forti, Oleksandr Frei, Masaki Fukunaga, Jayne Y. Hehir‐Kwa, Manon H. J. Hillegers, Per Hoffmann, Georg Homuth, Neda Jahanshad, Sanne Koops, Kuldeep Kumar, Masataka Kikuchi, Stephanie Le Hellard, Costin Leu, Robin M Murray, Terje Nærland, Lars Nyberg, Roel A. Ophoff, G Bruce Pike, Sigrid B. Sando, Jean Shin, Elena Shumskaya, Sanjay M. Sisodiya, Vidar M. Steen, Alexander Teumer, Anne Uhlmann, Margaret J. Wright, for the ENIGMA 22q11.2 Deletion Syndrome Working Group, Kevin M. Antshel, Linda E. Campbell, Nicolas A. Crossley, T. Blaine Crowley, Eileen Daly, Ania M. Fiksinski, Jennifer K. Forsyth, Wanda Fremont, Naomi J. Goodrich‐Hunsaker, Maria Gudbrandsen, Rachel K. Jonas, Wendy R. Kates, Amy Lin, Kathryn L. McCabe, Hayley Moss, Declan G. Murphy, Kieran C. Murphy, Michael J. Owen, Kosha Ruparel, Tony. J. Simon, Therese van Amelsvoort, Jacob A. S. Vorstman

**Affiliations:** ^1^ Department of Medical Genetics Oslo University Hospital Oslo Norway; ^2^ Norwegian Centre for Mental Disorders Research (NORMENT), Division of Mental Health and Addiction Oslo University Hospital and University of Oslo Oslo Norway; ^3^ KG Jebsen Centre for Neurodevelopmental Disorders University of Oslo Oslo Norway; ^4^ Imaging Genetics Center Mark and Mary Stevens Neuroimaging and Informatics Institute, Keck School of Medicine, University of Southern California Marina del Rey California USA; ^5^ School of Mental Health and Neuroscience, Faculty of Health, Medicine and Life Sciences Maastricht University Maastricht The Netherlands; ^6^ Semel Institute for Neuroscience and Human Behavior, Departments of Psychiatry and Biobehavioral Sciences and Psychology University of California Los Angeles Los Angeles California USA; ^7^ Department of Mental Health Veterans Affairs Greater Los Angeles Healthcare System, Los Angeles California USA; ^8^ NORMENT, Institute of Clinical Psychiatry University of Oslo Oslo Norway; ^9^ Department of Psychiatric Research Diakonhjemmet Hospital Oslo Norway; ^10^ Department of Clinical Neuroscience Karolinska Institutet Stockholm Sweden; ^11^ Institute of Neuroscience and Medicine (INM‐1) Research Centre Jülich Jülich Germany; ^12^ Cecile and Oskar Vogt Institute for Brain Research, Medical Faculty University Hospital Düsseldorf, Heinrich‐Heine‐University Düsseldorf Düsseldorf Germany; ^13^ Department of Child and Adolescent Psychiatry Institute of Psychiatry and Mental Health, Hospital General Universitario Gregorio Marañon, IsSGM, Universidad Complutense, School of Medicine Madrid Spain; ^14^ Centro Investigación Biomédica en Red de Salud Mental (CIBERSAM) Madrid Spain; ^15^ Mathematics and Statistics Murdoch University Perth Western Australia Australia; ^16^ Department of Psychiatry Marqués de Valdecilla University Hospital, Valdecilla Biomedical Research Institute (IDIVAL) Santander Spain; ^17^ Department of Psychiatry and Neuropsychology Maastricht University Maastricht The Netherlands; ^18^ Department of Radiology and Nuclear Medicine VU University Medical Center Amsterdam The Netherlands; ^19^ Clinical Genetics Research Program Centre for Addiction and Mental Health Toronto Ontario Canada; ^20^ Dalglish Family 22q Clinic for Adults with 22q11.2 Deletion Syndrome, Toronto General Hospital University Health Network Toronto Ontario Canada; ^21^ Department of Psychiatry University of Toronto Toronto Ontario Canada; ^22^ Department of Biological Psychology Vrije Universiteit Amsterdam Amsterdam The Netherlands; ^23^ Amsterdam Public Health (APH) Research Institute Amsterdam UMC Amsterdam The Netherlands; ^24^ Institute of Diagnostic Radiology and Neuroradiology University Medicine Greifswald Greifswald Germany; ^25^ Child Health Evaluative Sciences The Hospital for Sick Children Research Institute Toronto Ontario Canada; ^26^ Tri‐institutional Center for Translational Research in Neuroimaging and Data Science (TReNDS) Georgia State, Georgia Tech, Emory Atlanta Georgia USA; ^27^ Institute for Anatomy I Medical Faculty & University Hospital Düsseldorf, University of Düsseldorf Düsseldorf Germany; ^28^ Institute of Medical Genetics and Pathology University Hospital Basel Basel Switzerland; ^29^ Department of Biomedicine University of Basel Basel Switzerland; ^30^ Department of Psychosis Studies Institute of Psychiatry, Psychology and Neuroscience, King's College London London United Kingdom; ^31^ Department of Forensic and Neurodevelopmental Sciences The Sackler Institute for Translational Neurodevelopmental Sciences, Institute of Psychiatry, Psychology and Neuroscience, King's College London United Kingdom; ^32^ HU Virgen del Rocio, IBIS, Universidad de Sevilla, CIBERSAM Sevilla Spain; ^33^ MRC Centre for Neuropsychiatric Genetics and Genomics, Division of Psychological Medicine and Clinical Neurosciences Cardiff University Cardiff United Kingdom; ^34^ Center for Multimodal Imaging and Genetics University of California San Diego La Jolla California USA; ^35^ Department Radiology University of California San Diego La Jolla California USA; ^36^ Department of Psychological Medicine Institute of Psychiatry, Psychology and Neuroscience, King's College London London United Kingdom; ^37^ Faculty of Health Queensland University of Technology (QUT) Brisbane Queensland Australia; ^38^ NORMENT, Department of Clinical Science University of Bergen Bergen Norway; ^39^ Cardiff University Brain Research Imaging Centre (CUBRIC) Cardiff United Kingdom; ^40^ Center for Neuroimaging, Genetics and Genomics School of Psychology, NUI Galway Galway Ireland; ^41^ LREN, Centre for Research in Neuroscience, Department of Neuroscience University Hospital Lausanne and University Lausanne Lausanne Switzerland; ^42^ Neurology Department Max‐Planck Institute for Human Brain and Cognitive Sciences Leipzig Germany; ^43^ MIND Institute and Department of Psychiatry and Behavioral Sciences University of California Davis Davis California USA; ^44^ Division of Psychological and Social Medicine and Developmental Neurosciences Faculty of Medicine, TU Dresden Dresden Germany; ^45^ Department of Pediatrics Perelman School of Medicine at the University of Pennsylvania Philadelphia Pennsylvania USA; ^46^ Department of Psychology University of Oslo Oslo Norway; ^47^ Department of Psychology Bjørknes College Oslo Norway; ^48^ Language and Genetics Department Max Planck Institute for Psycholinguistics Nijmegen The Netherlands; ^49^ Donders Institute for Brain, Cognition and Behaviour Radboud University Nijmegen The Netherlands; ^50^ Psychiatric and Neurodevelopmental Genetics Unit Center for Genomic Medicine, Massachusetts General Hospital Boston Massachusetts USA; ^51^ Department of Psychiatry, Massachusetts General Hospital Harvard Medical School Boston Massachusetts USA; ^52^ Tommy Fuss Center for Neuropsychiatric Disease Research Boston Children's Hospital Boston Massachusetts USA; ^53^ Department of Psychiatry Harvard Medical School Boston Massachusetts USA; ^54^ German Center for Neurodegenerative Diseases (DZNE) Site Rostock/Greifswald Greifswald Germany; ^55^ Department of Psychiatry and Psychotherapy University Medicine Greifswald Greifswald Germany; ^56^ Department of Psychiatry University of Pennsylvania Philadelphia Pennsylvania USA; ^57^ Youth Suicide Prevention, Intervention and Research Center Children's Hospital of Philadelphia Philadelphia Pennsylvania USA; ^58^ Medical Imaging Research Center, Department of Biomedical Engineering Illinois Institute of Technology Chicago Illinois USA; ^59^ Department of Biomedicine University of Bergen Bergen Norway; ^60^ Division of Psychiatry Haukeland University Hospital Bergen Norway; ^61^ Department of Neuromedicine and Movement Science, Faculty of Medicine and Health Sciences Norwegian University of Science and Technology Trondheim Norway; ^62^ Department of Radiology and Nuclear Medicine St. Olavs Hospital Trondheim Norway; ^63^ Department of Psychiatry and Biobehavioral Sciences University of California Los Angeles Los Angeles California USA; ^64^ Department of Pathology of Mental Diseases National Institute of Mental Health, National Center of Neurology and Psychiatry Tokyo Japan; ^65^ Department of Psychiatry Osaka University Graduate School of Medicine Osaka Japan; ^66^ Personalized Healthcare Analytics Genentech, Inc. South San Francisco California USA; ^67^ Department of Psychology Yale University New Haven Connecticut USA; ^68^ Department of Psychiatry Yale University New Haven Connecticut USA; ^69^ Department of Psychiatry, UMC Utrecht Brain Center, University Medical Center Utrecht Utrecht University Utrecht The Netherlands; ^70^ Department of Psychiatry University of Pittsburgh Pittsburgh Pennsylvania USA; ^71^ Department of Psychiatry Boston Children's Hospital Boston Massachusetts USA; ^72^ Semel Institute for Neuroscience and Human Behavior University of California Los Angeles Los Angeles California USA; ^73^ School for Mental Health and Neuroscience Maastricht University Maastricht The Netherlands; ^74^ Neuroscience and Mental Health Research Institute Cardiff University Cardiff United Kingdom; ^75^ Computer Science Georgia State University Atlanta Georgia USA; ^76^ Department of Biological and Medical Psychology University of Bergen Bergen Norway; ^77^ Facultad de Psicología Universidad Autónoma de Madrid Madrid Spain; ^78^ Centre for Healthy Brain Ageing (CHeBA), School of Psychiatry, Faculty of Medicine University of New South Wales Sydney New South Wales Australia; ^79^ Neuroscience Research Australia Sydney New South Wales Australia; ^80^ Division of Human Genetics Children's Hospital of Philadelphia Philadelphia Pennsylvania USA; ^81^ Division of Human Genetics and 22q and You Center Children's Hospital of Philadelphia Philadelphia Pennsylvania USA; ^82^ Institute for Molecular Bioscience The University of Queensland Brisbane Queensland Australia; ^83^ Psychiatric Genetics QIMR Berghofer Medical Research Institute Brisbane Queensland Australia; ^84^ Department of Psychology, Faculty of Social Sciences University of Oslo Oslo Norway; ^85^ University of Lausanne Lausanne Switzerland; ^86^ Faculty of Health, Medicine and Life Sciences Maastricht University Maastricht The Netherlands; ^87^ Department of Radiology and Nuclear Medicine Maastricht University Medical Center Maastricht The Netherlands; ^88^ Sainte Justine Hospital Research Center University of Montreal, Montreal QC Canada; ^89^ Bloorview Research Institute Holland Bloorview Kids Rehabilitation Hospital Toronto Ontario Canada; ^90^ Departments of Psychology and Psychiatry University of Toronto Toronto Ontario Canada; ^91^ Translational Medicine, The Hospital for Sick Children Toronto Ontario Canada; ^92^ Bioinformatics Service, Nucleus University of Salamanca Salamanca Spain; ^93^ Biomedical Engineering Oregon Health and Science University Portland Oregon USA; ^94^ Centre for Lifespan Changes in Brain and Cognition, Department of Psychology University of Oslo Oslo Norway; ^95^ Psychiatric Imaging Group, MRC London Institute of Medical Sciences (LMS), Hammersmith Hospital Imperial College London London United Kingdom; ^96^ Center for Genetics and Genomics Facultad de Medicina, Clinica Alemana Universidad del Desarrollo Santiago Chile; ^97^ Center for Integrative Genomics University of Lausanne Lausanne Switzerland; ^98^ Service de Troubles du Spectre de l'Autisme Lausanne University Hospital Lausanne Switzerland; ^99^ Neuropsychiatric Institute The Prince of Wales Hospital Sydney New South Wales Australia; ^100^ Department of Radiology and Psychiatry University of Pennsylvania Philadelphia Pennsylvania USA; ^101^ School of Medical Sciences UNSW Sydney Sydney New South Wales Australia; ^102^ School for Mental Health and Neuroscience, Department of Psychiatry and Neuropsychology, Faculty of Health, Medicine and Life Sciences Maastricht University Maastricht The Netherlands; ^103^ Population Genomics, deCODE genetics/Amgen Reykjavik Iceland; ^104^ SA MRC Unit on Risk & Resilience in Mental Disorders, Department of Psychiatry and Neuroscience Institute University of Cape Town Cape Town South Africa; ^105^ PROMENTA Research Center, Department of Psychology University of Oslo Oslo Norway; ^106^ Neuroimaging Unit, Technological Facilities Valdecilla Biomedical Research Institute (IDIVAL), Santander Spain; ^107^ Faculty of Electrical and Computer Engineering University of Iceland, Reykjavik Iceland; ^108^ Social, Genetic and Developmental Psychiatry Centre Institute of Psychiatry, Psychology & Neuroscience, King's College London London United Kingdom; ^109^ School of Medicine University of Cantabria Santander Spain; ^110^ Department of Psychiatry The University of British Columbia Vancouver British Columbia Canada; ^111^ Faculty of Medicine University of Iceland Reykjavik Iceland; ^112^ NORMENT, Division of Mental Health and Addiction Oslo University Hospital Oslo Norway; ^113^ Department of Pediatrics University of Montreal, Montreal QC Canada; ^114^ Center for Neurobehavioral Genetics University of California Los Angeles Los Angeles California USA

**Keywords:** brain structural imaging, copy number variant, diffusion tensor imaging, evolution, genetics‐first approach, neurodevelopmental disorders, psychiatric disorders

## Abstract

The Enhancing NeuroImaging Genetics through Meta‐Analysis copy number variant (ENIGMA‐CNV) and 22q11.2 Deletion Syndrome Working Groups (22q‐ENIGMA WGs) were created to gain insight into the involvement of genetic factors in human brain development and related cognitive, psychiatric and behavioral manifestations. To that end, the ENIGMA‐CNV WG has collated CNV and magnetic resonance imaging (MRI) data from ~49,000 individuals across 38 global research sites, yielding one of the largest studies to date on the effects of CNVs on brain structures in the general population. The 22q‐ENIGMA WG includes 12 international research centers that assessed over 533 individuals with a confirmed 22q11.2 deletion syndrome, 40 with 22q11.2 duplications, and 333 typically developing controls, creating the largest‐ever 22q11.2 CNV neuroimaging data set. In this review, we outline the ENIGMA infrastructure and procedures for multi‐site analysis of CNVs and MRI data. So far, ENIGMA has identified effects of the 22q11.2, 16p11.2 distal, 15q11.2, and 1q21.1 distal CNVs on subcortical and cortical brain structures. Each CNV is associated with differences in cognitive, neurodevelopmental and neuropsychiatric traits, with characteristic patterns of brain structural abnormalities. Evidence of gene‐dosage effects on distinct brain regions also emerged, providing further insight into genotype–phenotype relationships. Taken together, these results offer a more comprehensive picture of molecular mechanisms involved in typical and atypical brain development. This “genotype‐first” approach also contributes to our understanding of the etiopathogenesis of brain disorders. Finally, we outline future directions to better understand effects of CNVs on brain structure and behavior.

## Graphical abstract

The enhancing neuroimaging genetics through meta‐analysis (ENIGMA) copy number variant (CNV) and 22q11.2 Working Groups focus on gaining insight into how rare genetic variants affect human brain development, cognition, and behavior. The two ENIGMA working groups have collated CNV and brain‐imaging data from numerous individuals, gathered by numerous international research centers, and analyzed this data with standardized processing and analysis pipelines. Future directions for the ENIGMA CNV and 22q11.2 working groups are to analyze CNVs with larger sample sizes and more imaging modalities to better understand how rare genetic variants affect the brain, and their clinical and behavioral consequences.

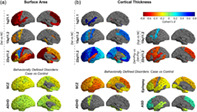

## 1 INTRODUCTION

Classical twin and family studies show that most complex human traits are moderately to highly heritable, including brain structure and function (Hilker et al., 2018; Jansen, Mous, White, Posthuma, & Polderman, 2015; Teeuw et al., 2019). Since 2009, the Enhancing NeuroImaging Genetics through Meta‐Analysis (ENIGMA) Consortium (Thompson et al., 2014; Thompson et al., 2020) and other large‐scale consortia such as Cohorts for Heart and Aging Research in Genomic Epidemiology (CHARGE) (Psaty & Sitlani, 2013) have made significant progress in identifying common genetic variants associated with variability in brain structure (Adams et al., 2016; Grasby et al., 2020; Hibar et al., 2015; Hibar et al., 2017; Knol et al., 2020; Satizabal et al., 2019; Stein et al., 2012) and function (Smit et al., 2018) through so‐called genome‐wide association studies (GWAS). The relatively common variants (genotyped in large numbers on single nucleotide polymorphism [SNP]) arrays in these studies are typically associated with minor variations in magnetic resonance imaging (MRI)‐derived brain measures, thus highlighting the polygenic nature of structural neuroanatomy. So far, our understanding of the biology including the impact of individual, single variants is limited. Therefore, identifying genetic variants with larger effects on MRI‐derived measures of brain structure or function may provide a path to help deduce molecular mechanisms contributing to brain development and diseases.

**FIGURE 1:**
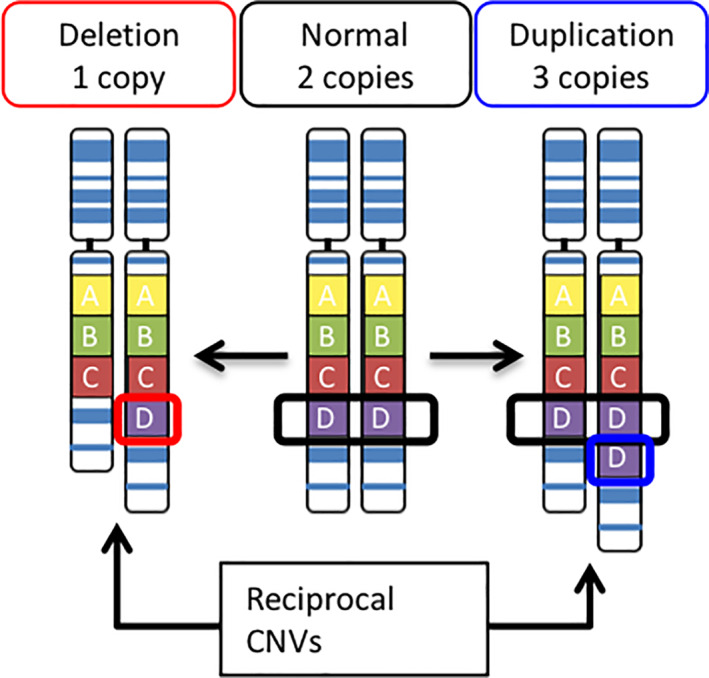
Copy number variants. CNV carriers may have a deletion (one copy of region D, red) or duplication (three copies of region D, blue) compared with the normal copy number (two copies of region D, black). Reciprocal CNVs are a deletion and duplication occurring at the same locus

Copy number variants (CNVs) (Figure 1) result from the deletion or duplication of a segment of the genome (Feuk, Marshall, Wintle, & Scherer, 2006) (a glossary of genetic terms is found in Table 1). CNVs represent a promising approach to study neurogenetic mechanisms shaping human behavior, cognition, and development. There are several rationales for this: certain rare, recurrent CNVs are associated with high risk (odds ratio up to 67.7) for a wide range of medical and behavioral consequences including brain disorders (Hastings et al., 2009) and some display large macroscopic effects on brain structure. The same CNV may confer elevated risk for several different (brain) disorders while reciprocal CNVs (Figure 1) at each end of the gene dosage response may be associated with the same disorder. Such clues gleaned from CNV research suggest that brain disorders are highly interlinked. Consequently, the study of rare CNV carriers may help us to understand the mechanisms behind not only rare isolated syndromes, but also of interrelated disorders, including the interaction between rare and common variants in shaping brain and disease as well as the intersection between somatic and brain disorders. This may allow us to identify both resilience and risk factors in common variants with potential to improve individual disease management.

**TABLE 1:** Glossary table

Term	Definition
Aneuploidy	The presence of an abnormal number of chromosomes in a cell. Examples are Down syndrome and monosomy X (Turner syndrome).
Anthropometric trait	A trait that describes body dimensions, such as head circumference, height, weight, girth, or body fat composition.
Array comparative genomic hybridization (aCGH)	A molecular cytogenetic method to detect copy number variants (CNVs) by comparing large fragments of DNA from a test individual to those from a reference sample.
Breakpoints (BP), chromosomal	A specific site of breakage, usually associated with a recurrent chromosomal abnormality. As in 16p11.2 distal CNV BP2‐BP3, where BP2‐BP3 refers to BP 2 to BP 3. For some CNVs, several low copy repeats (LCRs) in the region allow for multiple such BPs.
Copy number variant (CNV)	A type of structural genomic variation (Figure 1) that includes insertions, inversions, and translocations (Sharp, Cheng, & Eichler, 2006) in which segments of the genome are either deleted or duplicated. “Pathogenic” recurrent CNVs are of vastly different sizes and can span many genes (up to 90 for 22q11.2; McDonald‐McGinn et al., 2015) or just one (as in the case of *NRXN1* CNVs; Lowther et al., 2017). Differences in BPs within the same locus add to the complexity of CNVs (e.g., in the 16p11.2 or 1q21.1 regions). In addition to recurrent CNVs, numerous ultra‐rare nonrecurrent, “one‐hit,” or single CNVs may also disrupt normal function.
CNV ‐ naming	A CNV is named based on its locus, that is, its specific position on the chromosome. The shorter arm of a chromosome is termed the *p*‐arm (petite = French for small), while the longer arm is the *q*‐arm. For example, the 16p11.2:16 = chromosome 16; p = p‐arm; 11 = region 1, band 1; 2 = sub‐band 2. Distal and proximal are used when two CNVs are present at the same locus (e.g., the 16p11.2 distal and proximal CNVs)—Distal is situated farther away from the centre of the chromosome (called the centromere) than the proximal which is closer to the centromere.
de novo	A genomic variation that occurs spontaneously in the offspring and thus is not inherited from the parents.
Fluorescence in situ hybridization (FISH)	A targeted molecular cytogenetic method used to detect and localize a chromosomal deletion or duplication using fluorescent probes corresponding to the DNA sequence targeted.
Gene dosage effect	The relationship between the number of copies of a gene and, for example, gene expression or brain volume.
Gene dose response	The effect of altering the amount of genetic material in a region/the magnitude of the response of an organism to changes in gene presence.
Genome assembly/build	A reference genome assembly is a string of digital ATCG nucleotides representing the complete set of genes from an organism. It is assembled through a consensus of the genomes of different donors. The most recent human genome assembly, termed GRCh38 (also called “build 38”), was released in 2013 and is derived from 13 anonymous donors. Earlier human reference genome versions include: GRCh37 or hg19 (2009), NCBI36 or hg18 (2006), NCBI35 or hg17 (2004), and NCBI34 or hg16 (2003)
Genetics‐first approach	A strategy used in epidemiological studies to associate specific genotypes (such as a specific CNV) with apparent clinical phenotypes of a complex disease or trait. Also called “genotype‐first.”
Genotyping	The process of determining differences in the genetic make‐up (genotype) of an individual by examining the individual's DNA sequence using biological assays. The term is often used to refer to the identification of SNPs through (SNP) genotyping arrays.
Genetic heterogeneity	The same or similar phenotypes caused by different genetic mechanisms.
Idiopathic	Any disease or condition for which the cause is unknown.
Insertion	A structural variant that involves a mutation through the addition of genetic material to a chromosome.
Inversion	A structural variant in which a segment of a chromosome is reversed end to end.
Low copy repeats (LCRs)	Highly homologous sequence elements within the eukaryotic genome arising from segmental duplication and predisposing the genome to nonallelic homologous recombination (NAHR). LCRs mediate many of the chromosomal rearrangements that underlie genomic disorders by predisposition to recombination errors.
Multiplex ligation‐dependent probe amplification (MLPA)	A molecular cytogenetic method used to identify copy number variants. It is a variation of the multiplex polymerase chain reaction that permits amplification of multiple targets with only a single primer pair.
Nonallelic homologous recombination (NAHR)	A form of homologous recombination that occurs in two pieces of DNA that have similar sequences, often as a result of the presence of low copy repeats (LCRs). NAHR can occur within the same LCR or in an alternative LCR, and can result in a variety of chromosomal rearrangements, including deletion, duplication, translocation, and inversion. The presence of LCRs and resultant NAHR is believed to play a key role in molecular evolution in primates, as a mechanism involved in rapidly changing gene dosage (which may be advantageous) and even in the creation of new genes.
Noncarrier	In the context of CNVs, this is usually defined as an individual who does not carry the particular CNV being studied.
Penetrance	The proportion of people with a particular genotype/CNV who have any signs or symptoms of the disease.
Pleiotropy	The phenomenon whereby one allele (or a pair of alleles) influences multiple, independent phenotypes.
Polygenic trait	A phenotype that is influenced by multiple genetic variants at different genomic sites.
Rare CNV	Typically defined as a CNV with <1% frequency in the population.
Reciprocal CNVs	Deletions and duplications that occur at the same locus, usually flanked by LCRs.
Recurrent CNVs	CNVs that occur as spontaneous de novo events at the same sites in the genome repeatedly in unrelated individuals due to the presence of flanking low copy repeats, or LCRs) (Hastings, Lupski, Rosenberg, & Ira, 2009). In other words, they occur de novo in the first individual, and hence are not observed in the CNV carrier's parents but are potentially inherited in subsequent generations.
Single nucleotide polymorphism (SNP)	The substitution of a single base (A, T, C, or G) for another base at a specific genetic location that occurs in at least 1% of the population. A SNP may or may not have functional consequences on gene expression.
SNP genotyping array	DNA microarray used to detect SNPs within a population.
Somatic disease	A disease relating to the body, especially as distinct from the mind.
Translocation	A structural variant in which a portion of a chromosome breaks from its original location and reattaches to a different location in the genome.

**FIGURE 2:**
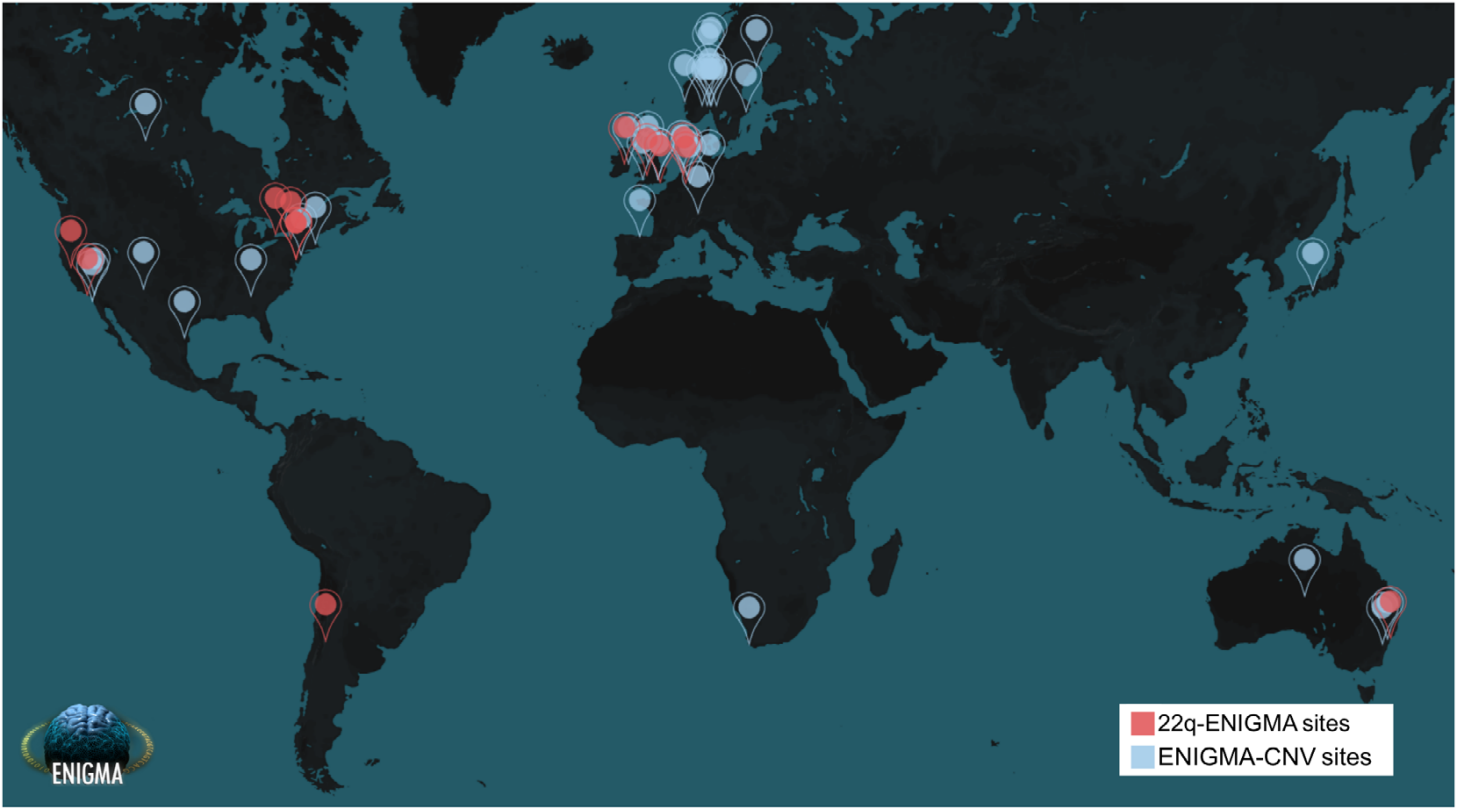
World map of the ENIGMA‐CNV and 22q‐ENIGMA WG study sites. A full list of participating cohorts and members for ENIGMA‐CNV and 22q‐ENIGMA may be found at the respective webpages: http://enigma.ini.usc.edu/ongoing/enigma‐cnv/enigma‐cnv‐co‐authors/ and http://enigma.ini.usc.edu/ongoing/enigma‐22q‐working‐group/22qwg/. Both working groups consist of international teams of clinicians, neuroscientists, engineers, bioinformaticians, statisticians, computer scientists, and geneticists who pool their resources to conduct large‐scale neuroimaging studies of CNVs

Despite their clinical relevance and evolutionary importance (Lauer & Gresham, 2019), effects of rare CNVs on human brain structure are poorly understood partially due to the rarity of these CNVs, which pose challenges in data collection. Several consortia including the 16p11.2 European consortium (Maillard et al., 2015) and Simons VIP/Searchlight (Qureshi et al., 2014) as well as individual projects (Meda, Pryweller, & Thornton‐Wells, 2012; Reiss et al., 2004; Stefansson et al., 2014; Ulfarsson et al., 2017) have addressed this. In addition, under the umbrella of ENIGMA, two groups—the ENIGMA 22q11.2 Deletion Syndrome Working Group (22q‐ENIGMA WG) and the ENIGMA‐CNV Working Group (ENIGMA‐CNV WG)—are devoted to increasing knowledge of the effect of CNVs on the brain. The 22q‐ENIGMA WG was founded in 2014 based on an extensive sample of 22q11.2 deletion carriers with brain MRI data (Figure 2). The ENIGMA‐CNV WG was formed in 2015 to study rare CNVs beyond the 22q11.2 locus and collated previously collected neuroimaging samples with genome‐wide individual genotyping (Figure 2). Both WGs aim to address some of the core limitations, especially those relating to low power and replicability, of prior brain imaging CNV studies and to foster collaborative discovery.

In this review, we focus on the work done by the ENIGMA WGs on CNVs. We first outline the significance of CNVs for elucidating genetic mechanisms underlying brain development and disease. We then describe the data collection, study design, and analytical methods used by the two WGs. Next, we review key findings of the 22q‐ENIGMA and ENIGMA‐CNV WGs on the 22q11.2, 16p11.2 distal, 15q11.2, and 1q21.1 CNVs and include results from other relevant work that has helped us to understand effects of CNVs on brain structure. We then discuss emerging principles that may govern how rare CNVs affect the brain. Finally, we summarize future plans to understand the neurobiology of CNVs for a broader range of brain phenotypes.

## 2 CNVS: HIGHLY RELEVANT FOR RISK FOR NEURODEVELOPMENTAL DISORDERS AND DRIVERS OF HUMAN BRAIN EVOLUTION

### 2.1 The role of CNVs in neurodevelopmental disorders

CNVs may account for up to 13% of the genome (Stankiewicz & Lupski, 2010), with the vast proportion being common across individuals and without any known negative effects (Iafrate et al., 2004). However, some CNVs can disrupt normal function in humans, causing psychiatric and neurodevelopmental disorders (NDs), somatic and neurological diseases, as well as cancer (Hastings et al., 2009). For instance, individuals with rare, recurrent CNVs are at much higher risk of NDs, including autism spectrum disorders (ASD), epilepsy, schizophrenia (SCZ), and intellectual disability (ID) (Kirov, Rees, & Walters, 2015) as well as Alzheimer's disease and other neurodegenerative diseases (Cervera‐Carles et al., 2016; Cuccaro, De Marco, Cittadella, & Cavallaro, 2017). De novo and inherited CNVs combined have been estimated to explain ~15% of neurodevelopmental disorder cases (Wilfert, Sulovari, Turner, Coe, & Eichler, 2017). Likewise, at least 9% of all ASD (Munnich et al., 2019) and 2.5% of SCZ cases carry a known pathogenic CNV (Rees et al., 2014). CNV carriers also have high rates of additional comorbid medical conditions (Crawford et al., 2018) and some display altered anthropometric traits (Mace et al., 2017; Owen et al., 2018). This high disease rate is often mirrored by reduced fecundity (Stefansson et al., 2014). Thus, altogether, a high impact CNV may represent a lifelong burden for the affected individuals and their caregivers, leading to substantial personal and societal costs.

The high odds ratio (>10) (Marshall et al., 2017) for neurodevelopmental disorders associated with specific CNVs is in contrast to the highest effect sizes identified for individual common genetic variants in SCZ (OR = 1.09; SCZ WG of the Psychiatric Genomics Consortium, 2014), bipolar disorder (OR = 1.13; Stahl et al., 2019), ASD (OR = 1.25; Grove et al., 2019), major depressive disorder (OR = 1.05; Wray et al., 2018) and attention deficit hyperactivity disorder (ADHD; OR = 1.12; Demontis et al., 2019). This has spurred considerable interest in studying CNVs as a genetics‐first approach to understand mechanisms of abnormal brain development as well as risk for disorders such as SCZ (Kirov et al., 2015), ASD (Stessman, Bernier, & Eichler, 2014) in addition to other medical comorbidities (Pierpont et al., 2018).

Such interest has been further encouraged by the diversity of CNVs: There are at least 93 known clinically relevant recurrent rare CNVs (Kendall et al., 2016), each with its own clinical profile/consequences (Girirajan et al., 2012; Rosenfeld & Patel, 2017). Some recurrent CNVs have moderate to small effects, for example, the more common 15q11.2 deletion, while others have large effects with near‐complete penetrance, such as the very rare Williams syndrome/7q11.23 deletion. Such high penetrance is positively correlated with the proportion of de novo occurrence in the population (Rosenfeld, Coe, Eichler, Cuckle, & Shaffer, 2013). In contrast, CNVs with small effects tend to be inherited more often, and may be identified in seemingly asymptomatic parents. Thus, different CNVs allow insight into different clinical risk profiles and their potential mechanisms.

Likewise, a specific CNV lacks diagnostic specificity and offers hugely diverse pleiotropic outcome. For instance, the same CNV may be associated with congenital defects, SCZ, ASD, ID, epilepsy, or early‐onset Parkinson's disease (Bijlsma et al., 2009; Shen et al., 2011; Stefansson et al., 2014; Tabet et al., 2012) as exemplified by the 22q11.2 deletion syndrome (22q11DS), which has been associated with all the above conditions (Boot et al., 2018; Butcher et al., 2013; Gudmundsson et al., 2019; Marshall et al., 2017). Furthermore, CNVs may lead to multiple disorders in the same individual (known as multimorbidity). For example, individuals with 22q11DS who have a psychiatric disorder are at increased risk for other psychiatric disorders, as well as motor coordination problems (Cunningham et al., 2018) and sleep problems (Moulding et al., 2020). Thus, few traits show evidence of genotypic specificity (Chawner et al., 2019; Cunningham, Hall, Einfeld, Owen, & van den Bree, 2020; Girirajan et al., 2012; Rosenfeld & Patel, 2017).

Harmful effects of CNVs may be partially explained by altered expression of genes in the affected region due to the difference in gene copy number, leading to higher or lower transcription levels (Hastings et al., 2009). This phenomenon is sometimes referred to as the “gene dosage effect” or “dose response per copy number.” CNVs can also modulate expression of genes outside of the region deleted or duplicated, either by addition or removal of regulatory elements, or by modifications of the 3D structure of the genome (Spielmann, Lupianez, & Mundlos, 2018). Thus, CNVs is a means for studying the effects of gene dosage alterations for many genes at a time and how these shape neurodevelopmental disease and brain structure.

The 22q11.2 region is an interesting region in this regard as it displays dose response with regard to SCZ risk: the deletion is associated with increased risk (Schneider et al., 2014) but the duplication appears to be associated with decreased risk (Marshall et al., 2017; Rees et al., 2014). In contrast, reciprocal CNVs may also carry risk for related disorders. For instance, the 22q11.2 deletion and duplication both confer high risk of ADHD (Gudmundsson et al., 2019), Likewise, the reciprocal 16p11.2 distal and proximal (Loviglio et al., 2016; Niarchou et al., 2019), 1q21.1 distal (Bernier et al., 2016; Mefford et al., 2008) and 22q11.2 loci (Lin et al., 2020) all confer risk of ASD. In this context, it is noteworthy, that population‐based studies overall suggest milder effects of duplication (vs. deletion) CNVs on cognition (Männik et al., 2015), which could suggest differences in the severity of, for example, ID in the reciprocal CNVs. Thus, CNVs allow investigations into how reciprocal CNVs at each end of the gene dosage response can cause both a “gene dose response” for disease risk but also similar disease risk.

The ultimate phenotype of a CNV likely depends on both environmental impacts and genetic background (Cleynen et al., 2020; Huguet et al., 2018; Kirov et al., 2014). Such influencing genetic factors likely include protective or disease‐enhancing genes located within the CNV region, or elsewhere in the genome. Educational attainment as a proxy for parental intelligence, for example, seems to modulate intellectual impairment related to a 22q11.2 deletion (Klaassen et al., 2016), indicating interplay of the CNV with common variants. The interactions between genetic factors as well as the environment will be key to a better understanding of CNV‐mediated disease risk. Investigations of interactions between CNVs and polygenic risk score as a proxy for common variants have already been initiated in disorders such as SCZ (Bergen et al., 2018; Davies et al., 2020; Tansey et al., 2016) and ADHD (Martin, O'Donovan, Thapar, Langley, & Williams, 2015). Thus, studies of CNV carriers may help disentangle the effects of the combination of rare and common variants as well as environment in shaping neurodevelopmental disease risk.

### 2.2 The role of CNVs in brain evolution

Changes in DNA—including CNVs—occur naturally and are a part of the evolutionary process and adaptation (Hastings et al., 2009) in all living organisms including animals and plants (Lauer & Gresham, 2019). Gene duplications provide a driving force in evolution (Bailey & Eichler, 2006) by allowing for the adaptation of new gene copies while maintaining the function of the old gene copy (Innan & Kondrashov, 2010). Even so, they also put the next generation at risk for re‐arrangements due to the presence of low copy repeats (LCRs), long clusters of related gene sequences with high sequence identity, that arise via duplication (Harel & Lupski, 2018). Interestingly, in the human and great ape lineage, there are proportionately more deletions and duplications observed in comparison to other mammals (Hahn, Demuth, & Han, 2007).

Some of these duplications have been hypothesized to be major driving forces in the rapid evolution of the human and great ape lineages (Dennis & Eichler, 2016) including brain enlargement and have given rise to entirely new human‐specific genes with novel characteristics. Examples include *SRGAP2* (three copies in humans, one in nonhuman primates) (Dennis et al., 2012), *NOTCH2NL* (three–four copies in humans, one in primates) (Fiddes et al., 2018; Suzuki et al., 2018) and *BOLA2* (Giannuzzi et al., 2019). The *NOTCH2NL* and *SRGAP2* genes are particularly interesting in the context of brain development: The *NOTCH2NL* genes confer delayed neuronal differentiation and increased progenitor self‐renewal (Fiddes et al., 2018; Suzuki et al., 2018), their occurrence coincides with a time just before or during the early stages of the expansion of the human cortex and they have thus been hypothesized to have contributed to the rapid evolution of the human neocortex. Likewise, transient overexpression of *SRGAP2C* in culture and in vivo leads to human‐specific features, including neoteny of dendritic spine maturation, promotion of longer spines at a greater density, and sustained radial migration in the developing mouse neocortex. Thus, duplications in human evolution appear to have shaped the formation of the human brain.

To date, discoveries on CNV‐related phenotypes have been hindered by the low frequency of each single pathogenic CNV in the general population (from 1 in ~400 to 1 in ~50,000 for recurrent CNVs; Kendall et al., 2016; Stefansson et al., 2014), making it challenging to collect sufficiently large, well‐powered samples. Even so, new technologies have moved the field forward considerably during the last 10 years.

## 3 NEW TECHNOLOGY—BIG DATA ANALYTICS IN GENETICS AND IMAGING

### 3.1 Genotyping and CNV calling

Among the earliest genetic syndromes to be detected were those caused by aneuploidies, such as trisomy 21 (Down's syndrome) and monosomy X (Turner syndrome). Testing for such genetic syndromes was incorporated into clinical practice in the 1950s and involved counting the number of chromosomes per cell, a technique known as karyotyping (Durmaz et al., 2015). Since then, a number of techniques including targeted fluorescence in situ hybridization (FISH), genome‐wide array comparative genomic hybridization (aCGH) and SNP arrays have allowed detection of smaller aberrations including CNVs down to ~10 kb (Nowakowska, 2017).

In 2004, two landmark studies (Iafrate et al., 2004; Sebat et al., 2004) showed that submicroscopic variations (<500 kb in size) in DNA copy number are widespread across the human genome. In the last 10–15 years, it has become possible to obtain genome‐wide CNV “calls” for many individuals through massive population‐scale SNP genotyping followed by demanding computational analyses. Likewise, clinical investigations and detection have become standard for some disorders. These new developments in technology have been vital for the increased knowledge of CNV carriers obtained in recent years.

### 3.2 Neuroimaging as a tool to study CNV effects on the brain

For some time, clinical observations have indicated characteristic macroscopic brain alterations in specific CNV carriers: reciprocal carriers of 16p11.2 and 1q21.1 distal CNVs (Brunetti‐Pierri et al., 2008) display macro‐ and microcephaly, respectively, and 17p13.3 deletion carriers (causing Miller‐Dieker syndrome) present with lissencephaly (smooth cortex because of lack of development of gyri and sulci) (Blazejewski, Bennison, Smith, & Toyo‐Oka, 2018). Thus, clinical data indicate that rare CNV carriers can teach us valuable lessons about brain development.

More detailed mapping through MRI—a reliable, noninvasive technique for mapping macro brain structure and functional consequences—have dived deeper and shown wide‐reaching phenotypic impacts of CNVs with substantial structural and functional alterations in the brain; for example, 22q11.2 deletions and duplications (Lin et al., 2017; Sun et al., 2018), 7q11.23 deletion (Fan et al., 2017; Meda et al., 2012), 15q11.2 (Silva et al., 2018; Stefansson et al., 2014; Ulfarsson et al., 2017; van der Meer et al., 2020) and 16p11.2 proximal (Maillard et al., 2015; Martin‐Brevet et al., 2018; Qureshi et al., 2014) and 16p11.2 distal CNVs (Sonderby et al., 2018). The 22q‐ENIGMA and ENIGMA‐CNV WGs have contributed significantly to this effort, by combining already collected cohorts of clinically ascertained samples on 22q11.2DS (Ching et al., 2020; Sun et al., 2018), as well as primarily non‐clinically ascertained samples for brain CNV research, so far publishing on 16p11.2 distal, 15q11.2 BP1‐BP2 and 1q21.1 distal (Sønderby et al., 2021; Sonderby et al., 2018; van der Meer et al., 2020).

### 3.3 ENIGMA‐standardized image processing

A prerequisite for large imaging studies is the standardization of approaches. The publicly available ENIGMA imaging processing and analysis protocols make it possible to consistently extract brain measures, and perform quality assessment and statistical modeling across many international research centers (http://enigma.ini.usc.edu/protocols).

ENIGMA processing pipelines applied in the ENIGMA‐CNV (point 1) and 22q‐ENIGMA WG (points 1–3) include:

1. **ROI brain measures**: Subcortical and cortical regions of interest (ROI) measures are extracted with FreeSurfer software (Fischl et al., 2002).
  - FreeSurfer subcortical volumes (eight gross volumetric features for both hemispheres) including thalamus, hippocampus, amygdala, caudate, putamen, pallidum, nucleus accumbens, lateral ventricles, and estimated intracranial volume (ICV) (as measured in the ENIGMA2 GWAS; Hibar et al., 2017).
  - Global and regional cortical thickness and cortical surface area measures (34 features for each hemisphere for both) based on the Desikan–Killiany atlas (Desikan et al., 2006; as measured in the ENIGMA3 cortical GWAS; Grasby et al., 2020).
2. **Vertex‐wise brain shape measures**: ENIGMA Subcortical Shape and FreeSurfer protocols are used to derive local thickness and surface area measures across cortical and subcortical structures.
  - Subcortical vertex‐wise shape modeling uses the ENIGMA Shape Analysis Pipeline to more finely map the spatial distribution of volumetric alterations across subcortical structures. The method derives local thickness and surface area expansion/contraction metrics for up to 2,502 vertices along the aforementioned subcortical ROIs, mapping potentially complex morphometric alterations (Ching et al., 2020).
  - Cortical thickness and surface area metrics extracted with FreeSurfer across tens of thousands of cortical vertices provides fine mapping of CNV‐related subregional cortical alterations (Sun et al., 2018).
3. **Diffusion‐weighted imaging and white matter microstructure**: The ENIGMA DTI protocol uses the tensor model and standardized DTI template to calculate fractional anisotropy (FA), mean diffusivity (MD), radial diffusivity (RD), and axial diffusivity (AD) in the Tract‐Based Spatial Statistics (TBSS) framework (Jahanshad et al., 2013; Smith et al., 2006); values for each measure are averaged along the skeleton of each ROI from the Johns Hopkins University White Matter Atlas (JHU‐ICBM‐DTI‐81) and analyzed in brain space (Mori et al., 2008). 18–25 ROIs are typically included (Kochunov et al., 2020).

These standardized feature extraction pipelines lead to more unbiased investigations of brain metrics, in that they are consistently applied across many data sets and cohorts. This approach improves upon traditional meta‐analyses, which often attempt to combine published effect sizes derived from different processing and analysis protocols. By pooling data derived using standard image processing pipelines in a coordinated effort, the ENIGMA‐CNV and 22q‐ENIGMA WG studies boost statistical power by incorporating data sets that may have been underpowered to detect brain effects on their own. The standardization of protocols, now being applied in large prospective studies such as UK Biobank (Alfaro‐Almagro et al., 2018), allows large‐scale comparison of brain measures and profiles of disease effects across studies to better characterize common and distinct brain signatures across CNVs and major brain disorders from independently collected study samples.

## 4 THE 22Q‐ENIGMA WG: DEEP DIVE INTO A HIGHLY PENETRANT GENETIC RISK FACTOR FOR PSYCHOSIS

22q11DS is a prominent example of a highly penetrant, recurrent CNV for which detailed phenotypic data has been collected in multiple cohorts worldwide (Gur et al., 2017). The main goals of the 22q‐ENIGMA WG are threefold: (a) map robust and reproducible multimodal brain markers of 22q11DS in large cohorts; (b) investigate how genetic and neuroanatomic variability relate to variability in phenotypic expression; and (c) determine convergence (and/or divergence) of neuroanatomical effects of high‐penetrance CNV versus behaviorally defined neuropsychiatric disorders.

**FIGURE 3:**
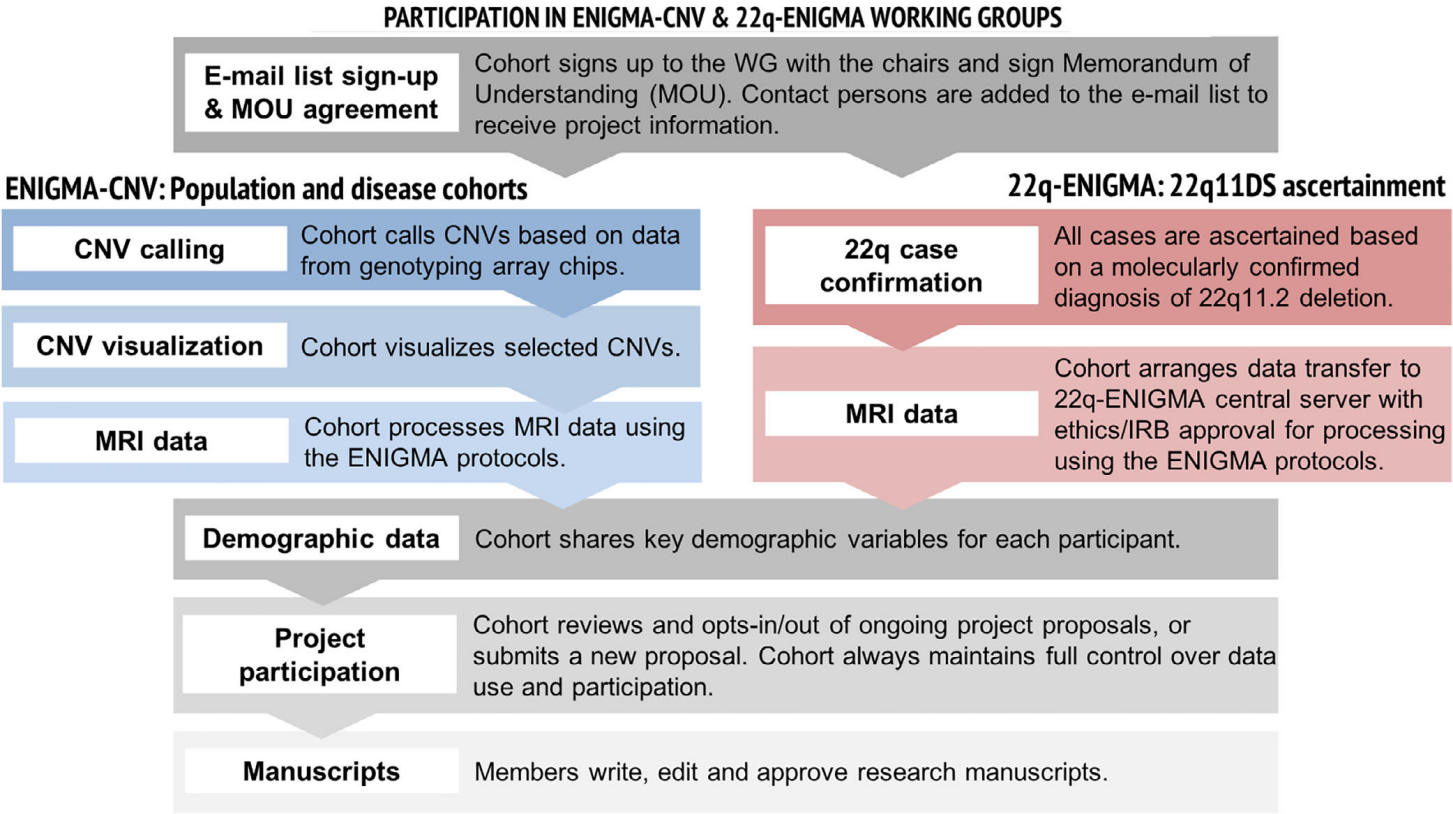
The overall procedure for participation in ENIGMA‐CNV and 22q‐ENIGMA

The 22q‐ENIGMA WG has built an international network of research programs and has centralized data from the largest available cohorts of 22q11.2 deletion carriers with brain imaging. The 22q‐ENIGMA WG consists of 12 international sites (Figure 2), and has analyzed data from over 533 individuals with molecularly confirmed 22q11.2 deletions and over 350 healthy controls. In addition, the UCLA lead site has collected 40 individuals with 22q11.2 duplications through a novel initiative. Age, sex, deletion size, IQ, history of psychosis and medication usage, along with structural and diffusion‐weighted imaging measures are collected and shared centrally for standardized processing and analysis (Figure 3).

### 4.1 Collection of CNV information

A molecularly confirmed diagnosis of 22q11.2 deletion is necessary for study inclusion. The most common deletion subtype, known as the LCR22A‐LCR22D or A‐D deletion, is found in ~85% of cases and involves the loss of ~2.6 megabases (Mb) of DNA. A smaller 1.5 Mb deletion—called the LCR22A‐LCR22B or A‐B deletion—is the next most common subtype, found in ~10% of cases (McDonald‐McGinn et al., 2015).

### 4.2 Demographic data harmonization

History of psychotic disorder is established by a trained mental health professional at each 22q‐ENIGMA site via a structured diagnostic interview, collateral information, and medical records. A cross‐site reliability procedure is conducted by two investigators to independently review representative cases from each site and to ensure diagnostic reliability across sites (Gur et al., 2017).

## 5 THE ENIGMA‐CNV WORKING GROUP: STANDARDIZED DATA COLLATION, PROCESSING AND ANALYSIS TO EMPOWER LARGE‐SCALE STUDIES

The primary goal of the ENIGMA‐CNV WG is to identify CNVs that significantly influence the brain globally and regionally to gain insight into the neurobiology of CNVs. The WG follows the main philosophy of the wider ENIGMA Consortium, which is to leverage existing legacy data sets to their full potential by combining samples using standardized processing. Notably, few of the research groups in ENIGMA‐CNV could have performed well‐powered CNV‐brain imaging studies on their own due to the low prevalence of individual CNVs.

### 5.1 Data collection and coordination

The large‐scale international nature of ENIGMA requires coordination of data originally collected with vastly different study designs, so initial analyses tend to be simple, followed by more complex analyses. From the beginning, ENIGMA‐CNV, rather than focusing on predefined selection of CNVs, chose a pragmatic approach driven by data availability. One key to success is a *unified approach* across studies for CNV calling, imaging analysis and quality control (Figure 3), given the differences in original cohort data collection and study design.

### 5.2 Standardized CNV calling and visualization across cohorts

The low frequency of recurrent CNVs makes a mega‐analysis approach preferable to the original ENIGMA meta‐analysis approach (Boedhoe et al., 2018). Given the lack of experience in genetic analysis, in particular CNV calling, for many participating cohorts, ENIGMA‐CNV first developed an easy‐to‐follow protocol for CNV calling. Many SNP genotyping arrays exist that vary in the number of SNPs included and their coverage of the genome. The often nonuniform distribution of tagged SNPs across the genome means that there may be limited coverage in regions with segmental duplications or complex CNVs (Carter, 2007). Consequently, larger CNVs (> 500 kb) can be reliably detected by microarrays from most platforms, whereas variability between platforms is greater for smaller CNVs (10–100 kb). A number of different CNV calling methods exist (Pinto et al., 2011). PennCNV (Wang et al., 2007), a widely used CNV calling software platform (Macé et al., 2016), was chosen since it accommodates a wide selection of SNP‐based arrays (e.g., *Affymetrix* and *Illumina*) and is user friendly and fast (Macé et al., 2016)—a key advantage at a time when the number of available samples increases at an unprecedented rate.

Most participating cohorts call CNVs themselves. Alternatively, the ENIGMA‐CNV WG does the calling on their behalf based on raw genotype information provided by the respective participating cohort. To address regulatory issues, the CNV calling protocol includes a de‐identification step. Following CNV calling, individual cohorts follow a CNV visualization protocol based on the iPsychCNV R package (https://github.com/mbertalan/iPsychCNV/). Finally, the WG analysts do the final, manual QC of the visualized CNVs to ensure harmonized CNV calls across cohorts.

Cohorts with smaller sample sizes should feel encouraged to join the ENIGMA‐CNV WGs as the number and nature of CNV carriers is unknown prior to CNV calling. As the project has developed, samples verified by alternative genotyping such as aCGH, Multiplex Ligation‐dependent Probe Amplification (MLPA) or FISH have also been included in the study. These typically constitute clinical samples, so the corresponding noncarriers (used as controls) have typically only been checked for presence or absence of the CNV of interest.

### 5.3 Demographic data

A minimal number of demographic metrics are collected, including age at brain scan, sex, diagnosis (if applicable), scanner site, and multidimensional scaling (MDS) factors (when available) from the analysis of population structure in the genome‐wide data.

### 5.4 Study and analysis design

In disease studies, controls are typically defined at the outset of the individual studies. This contrasts to ENIGMA‐CNV where controls, dubbed noncarriers, are individuals who do not carry the particular CNV being studied nor any other potentially pathogenic CNV (as defined by a precompiled list; Kendall et al., 2016). The latter allows a truly blinded sampling as neither the recruiters, nor the participants, knew CNV status at the time of the analysis except for the few clinically ascertained carriers.

For primary data analyses, ENIGMA‐CNV applies both a linear regression, to test the effect of the CNV per copy number of the region in question, that is, the dose response, and a *t* test to compare the pairs of groups (deletion or duplication vs. noncarriers or deletion vs. duplication carriers). Imaging data are adjusted for age at brain scan, sex, and scanner site—both with and without adjusting for ICV. The number of noncarriers in ENIGMA‐CNV is an order of magnitude larger than carriers. This provides the opportunity to perform an estimate of the effect of the CNVs in comparison to the overall population. Separate “sensitivity” analyses are performed including a matched analysis (matching each carrier with a noncarrier based on, e.g., age, sex, affection status, and ICV) as well as separate analyses that take into account ancestry information (MDS factors) and diagnoses (if known). These sensitivity analyses allow testing of the robustness of the results in selected subsets of the sample.

### 5.5 Overview of the ENIGMA‐CNV working groups

The ENIGMA‐CNV sample currently comprises a total of 38 cohorts (Figure 2) with genotyping and MRI data comprised of core ENIGMA‐CNV based on clinical (mostly case–control) and population studies as well as publicly available data sets (currently the UK Biobank) and represent a broad spectrum of CNVs (Table 2). Part of the strength of the ENIGMA‐CNV sample, compared to clinical CNV studies, is a higher proportion of high‐functioning CNV carriers given the high proportion of nonclinical “volunteer”/population samples (~70% of core ENIGMA‐CNV). This advantage comes with the downside of an under‐representation or absence of CNVs with high penetrance, such as individuals with Prader‐Willi/Angelman syndrome (15q11.2–13.2 deletion carriers), Sotos syndrome (5q35 deletion), the 22q11.2 deletion (Table 2) as well as severely functionally affected individuals carrying CNVs with a broad phenotypic spectrum, such as 1q21.1 distal, 16p11.2, and 15q11.2 CNVs. The under‐representation is partially compensated by fruitful collaboration with more clinically focused studies such as the 16p11.2 European consortium (Martin‐Brevet et al., 2018) and the Cardiff ECHO‐DEFINE/IMAGINE‐study (Chawner et al., 2019) and case–control studies, for example, epilepsy, SCZ, bipolar disorders, and ADHD. Consequently, ~10% of individuals in the core ENIGMA‐CNV sample has a known clinical diagnosis. Thus, the current ENIGMA‐CNV sample represents extensive parts of the phenotypic spectrum of CNV carriers. We continue to add samples to broaden the scope of the studies.

**TABLE 2:** Numbers of selected deletion (del) and duplication (dup) carriers and noncarriers (nc) in the current ENIGMA‐CNV sample including UK Biobank

CNVs of interest	Deletion carriers	Noncarriers	Duplication carriers
1q21 proximal (TAR)	22		69
1q21.1 distal	34		25
2p16.3 (NRXN1)	1		
3q29	1		
10q11.22–23	5		1
15q11.2	167		225
15q11.2‐q13			1
16p11.2 proximal	7		15
16p11.2 distal	10		12
16p11.2 distal proximal^a^	3		3
16p12.1	25		25
16p13.11	14		99
17p12	33		18
17q12	2		14
17q21.31			1
22q11.2, 2.6MB	2		22
Noncarriers		53,879	

^a^ The 16p11.2 distal proximal CNV spans both the distal and proximal region.

## 6 FINDINGS FROM THE 22Q‐ENIGMA AND ENIGMA‐CNV WGS

**FIGURE 4:**
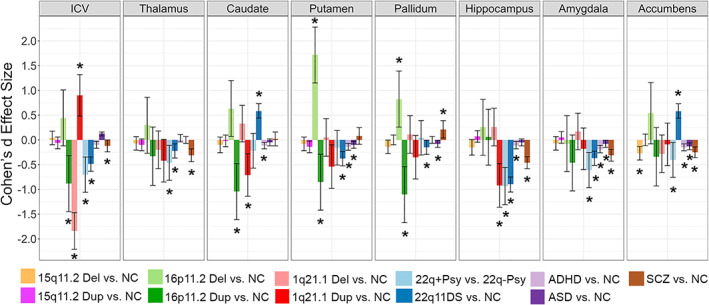
The subcortical findings from ENIGMA‐CNV, 22q‐ENIGMA and selected ENIGMA psychiatric working groups. Averaged left and right subcortical volume case versus non‐carriers (NC) Cohen's d effect size estimates for the ENIGMA SCZ (van Erp et al., 2016), ADHD (Hoogman et al., 2017), ASD (van Rooij et al., 2018), 22q11DS (Ching et al., 2020), 15q11.2 CNV (van der Meer, 2019), 16p11.2 distal CNV (Sønderby et al., 2018), and the 1q21.1 distal CNV (in review) studies. 22q+Psy vs. 22q‐Psy indicates a comparison from Ching et al. (2020) where a subset of individuals with 22q11.2 deletion syndrome with a history of psychosis were compared to a matched group of individuals with 22q11.2 deletion without a history of psychosis. Significant group differences are indicated by an asterisk (*); the plot includes vertical 95% confidence intervals

**FIGURE 5:**
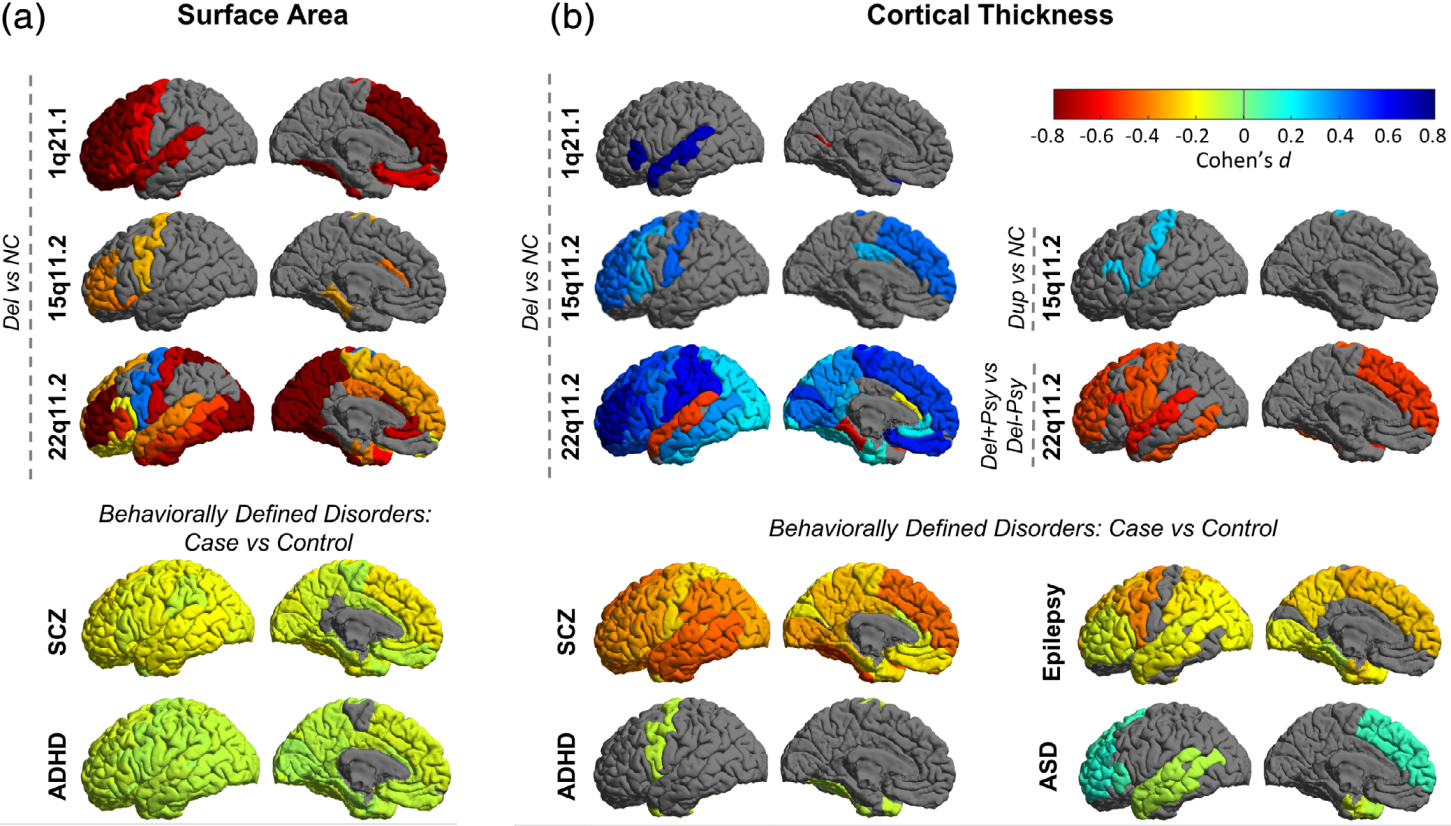
Cortical findings from the ENIGMA‐CNV, 22q‐ENIGMA, and selected ENIGMA psychiatric working groups. Copy number variant (CNV) analyses: for deletion or duplication carriers vs non‐carriers for the 15q11.2 CNVs (ICV‐corrected; van der Meer et al., 2019), 1q21.1 distal CNVs (ICV‐corrected; in review) and 22q11DS (Sun et al., 2018). 22q11DS results include 22q11DS psychosis deletion (Del+Psy) vs non psychosis deletion (Del‐Psy; left hemisphere shown). Behaviorally defined disorders analyses: Results are shown from case‐control studies from ASD's mega‐analysis (left hemisphere shown; van Rooij et al., 2018), all ages in ADHD combined (children, adolescents and adults; Hoogman et al., 2017), all types of epilepsies combined (left hemisphere shown; Whelan et al., 2018), and schizophrenia (SCZ; left hemisphere shown; van Erp et al., 2018). Only significant results are shown

To date, the 22q‐ENIGMA WG has published three peer‐reviewed studies on alterations of cortical, subcortical, and white matter structure, respectively (Ching et al., 2020; Sun et al., 2018; Villalon‐Reina et al., 2019). The ENIGMA‐CNV WG has also published three peer‐reviewed studies on the 16p11.2 distal CNV (Sonderby et al., 2018), the 15q11.2 CNV (van der Meer et al., 2020) and the 1q21.1 distal CNV (Sønderby et al., 2021) while two secondary projects are underway. Main results and comparisons with idiopathic disease can be seen in Figures 4 and 5. Analysis is ongoing for several more CNV regions.

### 6.1 22q11.2 deletion syndrome

#### 6.1.1 Cortical structure

The 22q‐ENIGMA WG analyzed the largest sample to date of brain images from individuals with 22q11DS, from 10 cohorts, including 474 individuals with 22q11DS (age = 18.2 ± 8.6 years; 46.9% female) and 315 matched, typically developing controls (age = 18.0 ± 9.2; 45.9% female) (Sun et al., 2018). Compared to controls, the 22q11DS group showed overall thicker cortex (left/right hemispheres: Cohen's *d* = 0.61/0.65) and widespread lower cortical surface area (left/right hemispheres: *d* = −1.01/−1.02), which was most prominent in parieto‐occipital and medial brain regions. Surface area decreases were less pronounced in deletion carriers with the smaller 1.5 Mb deletion (LCRA‐LCRB) compared to those with the larger, more typical ~2.6 Mb deletion (LCRA‐LCRD). This provided the first evidence of differential brain morphometry associated with 22q11DS deletion size. When applied to the cortical thickness and surface area measures, a machine learning method provided a high degree of accuracy (sensitivity 94.2%; specificity 93.3%) in classifying 22q11DS cases from healthy controls. Individuals with 22q11DS and a history of psychosis had a pattern of thinner cortex, particularly in frontotemporal regions (vs deletion carriers without a history of psychosis) that significantly overlapped with alterations reported in the largest study of cortical structure in idiopathic SCZ (van Erp et al., 2018). Importantly, the ENIGMA SCZ study (van Erp et al., 2018) and the 22q‐ENIGMA WG studies used the same image processing, quality control, and analysis protocols. These results lend further evidence that 22q11DS offers a biologically tractable framework to better understand the underlying mechanisms driving complex phenotypes such as psychosis.

#### 6.1.2 Subcortical structure

The 22q‐ENIGMA WG performed a mega‐analysis of subcortical volume and shape analysis (Ching et al., 2020) that included 533 individuals with 22q11DS and 330 matched healthy controls (HC; age: 6–56 years) from 11 study sites. Compared to HC, 22q11DS individuals had, on average, smaller bilateral hippocampal, putamen, amygdala and left thalamus volumes, and larger bilateral ventricle, caudate and accumbens volumes. However, a novel shape analysis technique revealed complex local morphometric differences between groups. Shape analysis also revealed regions of the hippocampus, caudate, accumbens, thalamus, and putamen that were less affected in individuals with the smaller (LCR22A‐LCR22B) deletion—the first time that subcortical morphometric variations have been tied to deletion size. Deletion carriers with a history of psychosis had smaller thalamic, hippocampal and amygdala volumes compared with matched carriers without psychosis. These alterations overlapped with the subcortical effects observed in the largest neuroimaging study of SCZ (van Erp et al., 2016), including smaller overall ICV, amygdala, hippocampal, and thalamic volumes. Furthermore, when compared to other ENIGMA subcortical psychiatric studies using the same image processing pipelines, subcortical volume alterations in 22q11DS‐associated psychosis were strongly correlated with case–control effects found in studies of major depressive disorder (Schmaal et al., 2016), bipolar disorder (Hibar et al., 2016) and obsessive compulsive disorder (OCD; Boedhoe et al., 2017), but not with subcortical patterns reported in studies of ASD (van Rooij et al., 2018) or ADHD (Hoogman et al., 2017). Overall, 22q11DS and 22q11DS‐associated psychosis effect sizes were larger than those found in all other ENIGMA studies of idiopathic psychiatric disorders (Figure 4). This lends credence to the idea that a genetics‐first approach may provide greater power to detect biomarkers by providing larger effect sizes than those associated with more common genetic variation (Medland et al., 2020, this issue).

#### 6.1.3 White matter structure

The first study of white matter microstructure from the 22q‐ENIGMA WG was a mega‐analysis of 334 deletion carriers and 260 healthy controls (age: 6–52 years) from 10 international sites (Villalon‐Reina et al., 2019). In the largest study of its kind, a widespread pattern of lower mean diffusivity, axial diffusivity and radial diffusivity and higher fractional anisotropy was detected in 22q11.2 deletion carriers compared to controls, with moderate to large effect sizes. Individuals with both 22q11DS and a history of psychosis displayed more pronounced abnormalities in diffusivity, which pointed to a pattern of white matter abnormalities that may reflect disrupted neurogenesis, particularly in outer layer cortical neurons. However, white matter alterations for individuals with 22q11DS and psychosis diverged from results reported in the largest study of idiopathic SCZ (1,963 SCZ and 2,359 healthy controls from Kelly et al., 2018), which used the same ENIGMA‐DTI processing and quality control pipelines. Whereas individuals with 22q11DS and a history of psychosis showed a general pattern of higher fractional anisotropy and lower diffusivity, people with idiopathic SCZ showed, on average, a pattern of lower fractional anisotropy and higher diffusivity, especially mean and radial diffusivity (Kuchonov et al., this issue). These opposing patterns in white matter variation (Bakker et al., 2016) stand in contrast to findings in cortical and subcortical gray matter, where brain alterations were largely convergent between 22q11DS psychosis and idiopathic SCZ. These findings suggest that different connectivity patterns in white matter may be associated with similar behavioral/clinical outcomes. Ongoing work using more advanced imaging protocols such as “multishell” diffusion MRI—combined with reliable biophysical models that estimate tissue microstructural properties—are being used to investigate the fiber tracts and cellular attributes leading to white matter vulnerabilities in 22q11DS (Villalon Reina et al., 2019; Villalon Reina, Nir, Kushan, Bearden, & Thompson, 2019).

### 6.2 Other CNVs

#### 6.2.1 16p11.2 distal CNV

This ENIGMA‐CNV study examined the impact of the 16p11.2 distal CNV on brain structure and function (Sønderby et al., 2018). The 16p11.2 distal CNV (BP2‐BP3, 28.7 to 28.9 Mb; hg18 genome assembly) predisposes carriers to psychiatric conditions including ASD and SCZ and had been associated with macro‐ and microcephaly in deletion and duplication carriers, respectively (Loviglio et al., 2016). It has a frequency of 0.02 and 0.04% for the deletion and duplication, respectively (Kendall et al., 2016; Smajlagić et al., 2020; Stefansson et al., 2014). The 16p11.2 distal CNV lies within a region with many LCRs that also give rise to the 16p11.2 proximal CNV (29.5–30.1, hg18, BP4–BP5) whose brain structural underpinnings have been studied in several studies (Cardenas‐de‐la‐Parra et al., 2019; Maillard et al., 2015; Martin‐Brevet et al., 2018; Qureshi et al., 2014). Both the 16p11.2 distal and proximal CNVs display a negative dose response for body mass index (BMI) (Mace et al., 2017; Owen et al., 2018) and head circumference (Jacquemont et al., 2011; Loviglio et al., 2016).

Based on 12 16p11.2 distal deletion, 12 duplication carriers and 6,882 noncarriers, we identified a negative dose response association of copy number, that is, greater volumes for deletions and lower volumes for the duplication, with ICV, caudate, pallidum and putamen volumes. The pallidum finding was replicated in a smaller sample from deCODE Genetics, Iceland. Further, the combined meta‐analysis of 15 16p11.2 distal deletion and 18 duplication carriers and 7,714 noncarriers identified a negative dose response on nucleus accumbens volume.

The minimal core segment of the 16p11.2 distal CNV is 200 kb in length and contains nine genes. A study in zebrafish found that only over‐expression of the *LAT* gene from the 16p11.2 distal region induced a decrease in cell proliferation in the brain with a concomitant microcephalic phenotype (Loviglio et al., 2017). *LAT* knockout mice also showed anatomical brain abnormalities (Loviglio et al., 2017) and brain regions expressing the highest levels of the *LAT* gene include basal ganglia (Hawrylycz et al., 2012), providing overlap with the brain structural changes identified in the ENIGMA‐CNV study. These findings provide converging evidence that LAT, an immune signaling adaptor, is a possible dosage‐dependent driver of the CNV‐associated brain phenotypes, including the alterations in the basal ganglia. These findings also fit well with a proposed role of the immune system in the development of psychiatric disorders such as SCZ (Khandaker et al., 2015). Notably, a recent GWAS on subcortical volumes identified a GWAS hit, rs1987471, for the caudate nucleus in the 16p11.2 distal region upstream of the *ATXNL2* gene (Satizabal et al., 2019), indicating that several genes in the interval may be involved in the brain structural changes.

#### 6.2.2 15q11.2 CNV

In this study, ENIGMA‐CNV targeted a more frequent CNV, the 15q11.2 CNV (BP1‐BP2, 20.3–20.8 Mb, hg18 genome assembly) with a population prevalence around 0.3% (Crawford et al., 2018; Stefansson et al., 2014). The deletion has unequivocally been associated with an increased risk for SCZ, (OR = 1.6; Marshall et al., 2017). Overall, the effect sizes on disease, cognitive and behavioral phenotypes on the duplication are absent or small: In fact, the duplication has not been clearly associated with psychiatric or neurodevelopmental disorders and its carriers perform on par with controls on cognitive tests (Abdellaoui et al., 2015; Kendall et al., 2019; Stefansson et al., 2014). In contrast, the deletion is associated with a reduction in IQ of ~4 points (Huguet et al., 2018; Jønch et al., 2019) while deletion carriers unaffected by psychiatric or neurodevelopmental disorders have an increased risk of dyslexia and dyscalculia (Stefansson et al., 2014). Reflecting these small effects, the vast majority of carriers are not clinically affected, and the deletion is inherited in >90% of the cases (Cox & Butler, 2015; Jønch et al., 2019; Mohan et al., 2019). Finally, 15q11.2 dosage has been reported to be associated with white matter alterations (Silva et al., 2019; Stefansson et al., 2014; Ulfarsson et al., 2017).

We assessed the association of the 15q11.2 CNV with cognition, and cortical and subcortical morphology in over 45,000 individuals from 38 cohorts, including the UK Biobank (203 individuals with a 15q11.2 deletion, 45,247 noncarriers, and 306 duplication carriers; van der Meer et al., 2020). We identified a clear pattern of widespread poorer cognitive performance, smaller surface area, and thicker cortices for deletion carriers compared to noncarriers and duplication carriers, particularly across the frontal lobe, anterior cingulate, and pre‐ and postcentral gyri.

The 15q11.2 region contains four evolutionarily highly conserved genes: *NIPA1*, *NIPA2*, *CYFIP1*, and *TUBGCP5* (Chai et al., 2003). The first three of these genes have known roles in neurodevelopment and contain polymorphisms associated with several brain disorders (Goytain, Hines, El‐Husseini, & Quamme, 2007; Goytain, Hines, & Quamme, 2008; Napoli et al., 2008; van der Zwaag et al., 2010). *CYFIP1* and *NIPA1* are highly expressed in the developing brain (van der Zwaag et al., 2010) and are key players in a number of processes contributing to brain plasticity, including axon outgrowth and dendritic spine formation (De Rubeis et al., 2013; Schenck et al., 2003; Wang, Shaw, Tsang, Reid, & O'Kane, 2007). Likewise, common CYFIP1 polymorphisms, that influence its expression levels, have been linked to variation in cortical surface area (Woo et al., 2016). Thus, the pattern of results fits well with known molecular functions of the genes in the 15q11.2 region, in particular *CYFIP1*, and suggests involvement of these genes in neuronal plasticity and cortical development.

#### 6.2.3 1q21.1 distal CNV

Carriers of the 1q21.1 distal CNVs (BP3‐BP4, 145–145.8 Mb, hg18 genome assembly) are at higher risk for several disorders including SCZ, ID, developmental delay, speech problems, ASD, motor impairment and epilepsy (Bernier et al., 2016; Chawner et al., 2019; Gourari, Schubert, & Prasad, 2018; Haldeman‐Englert & Jewett, 1993; Mefford et al., 2008) and separate risk for the duplication carriers for ADHD (Gudmundsson et al., 2019), bipolar disorder and major depressive disorder (Green et al., 2016; Kendall et al., 2019). The CNV has a frequency of 0.03% and 0.05% for the deletion and duplication, respectively, (Kendall et al., 2016; Stefansson et al., 2014). The 1q21.1 distal CNV has an effect on head circumference, as evident from a high prevalence of micro‐ and macrocephaly in deletion and duplication carriers, respectively (Bernier et al., 2016; Brunetti‐Pierri et al., 2008; Rosenfeld et al., 2012).

ENIGMA‐CNV systematically assessed brain structural MRI variation in 28 1q21.1 distal deletion and 22 duplication carriers and 37,088 noncarriers. We identified positive dosage effects of copy number on ICV and total cortical surface area, with the largest effects in frontal and cingulate cortices, and negative dosage effects on caudate and hippocampal volumes. The effects on subcortical volumes were also observed in a UK biobank exploratory study on six individuals with a 1q21.1 distal duplication (Warland, Kendall, Rees, Kirov, & Caseras, 2019). Carriers displayed distinctive deficits in cognitive tasks with intermediate decreases in duplication, and somewhat larger decreases in deletion carriers—the latter apparently mediated by the decrease in ICV and cortical surface area.

Despite the high effect sizes identified, at the time of writing, GWAS based on the hg19 genome assembly have not identified hits in the 1q21.1 genomic region for ICV (Adams et al., 2016; Knol et al., 2020), total cortical or regional surface area (Grasby et al., 2020; Hofer et al., 2020). Because of the many LCRs in the region (Brunetti‐Pierri et al., 2008; Sharp et al., 2006), assembly of the 1q21.1 region was faulty until version GRCh38 (Steinberg et al., 2014), likely inhibiting gene discovery and this may explain the current lack of GWAS hits in the region.

Given the different breakpoints of the 1q21.1 distal CNVs, estimates of the number of affected genes vary, but the core interval encompasses at least 12 protein‐coding genes including several human‐specific genes, such as *HYDIN2* (Dolcetti et al., 2013; Rosenfeld et al., 2012), *NOTCH2NL*s (Fiddes et al., 2018; Suzuki et al., 2018) and the DUF1220/Olduvai domain‐containing *NBPF*‐encoding genes. The recently characterized *NOTCH2NL* genes are particularly interesting in the context of brain development, and as candidates for a dosage‐dependent amplifier of the CNV‐associated brain phenotypes. They are absent in humans' closest living relatives—nonhuman primates—and confer delayed neuronal differentiation and increased progenitor self‐renewal (Fiddes et al., 2018; Suzuki et al., 2018)—in line with the radial unit hypothesis of cortical development (Rakic, 1995). A neurodevelopmental effect on cell proliferation fits well with the overall directional effect of this CNV on cortical surface area and ICV.

### 6.3 Summary and implications of the findings

A common finding across all four CNVs studied by the 22q11‐ENIGMA and ENIGMA‐CNV WGs is the presence of a gene dosage response on several brain structures (Sønderby et al., 2021; Lin et al., 2017; Sønderby et al., 2018; van der Meer et al., 2020) whose direction may differ between CNVs: carriers of 16p11.2 CNVs display a negative dose response on ICV, while individuals with 1q21.1 CNVs show a positive dose response. Likewise, a dose response on subcortical volumes may be in the same direction (e.g., caudate in 16p11.2 distal) or opposite direction (e.g., caudate in 1q21.1 distal) as that of a macroscopic effect on ICV. Likewise, a dose response may not span both reciprocal CNVs—for example, nucleus accumbens is only altered in 15q11.2 deletion carriers, not in the duplication.

Many CNV carriers overlap in terms of symptoms and susceptibility to disease. Even reciprocal CNVs at each end of the gene dose response can cause both a “gene dose response” for disease risk (22q11.2 and SCZ, Marshall et al., 2017) but also similar disease risk (16p11.2 proximal and distal, 22q11.2, and 1q21.1 distal) for ID and ASD (Chawner et al., 2019). The general rule seems to be that the effect on brain structure fits an additive model for gene dosage formed by, for example, gene expression, whereas an inverted U‐shaped effect curve is observed for the phenotype (Deshpande & Weiss, 2018).

Importantly, brain structural findings in CNV carriers appear to overlap, to some extent, with patterns of brain alterations found in several major brain disorders including ADHD (Hoogman et al., 2017), ASD (van Rooij et al., 2018), SCZ (van Erp et al., 2016), bipolar disorder (Hibar et al., 2016), major depressive disorder (Schmaal et al., 2016), and epilepsy (Whelan et al., 2018). However, several CNVs clearly have effect sizes far greater than those of the idiopathic diseases (Figures 4 and 5). Likewise, so far, it is difficult to find a direct pattern in the overlap between known disease susceptibility for CNV carriers and brain structural effects—both in terms of specificity (actual overlap) and effect sizes. Thus, this makes it increasingly evident that vastly different brain alterations—for example, large macroscopic effects (e.g., in 16p11.2 and 1q21.1 CNVs) as well as small subtle effects (e.g.,15q11.2 CNVs)—can lead to similar phenotypes, underlining the heterogeneity in brain structure within diseases such as SCZ and a putative potential to stratify based on brain structure within specific diseases. Improved understanding of these types of relationships may prove important for understanding disease susceptibility and outcome.

## 7 ONGOING PROJECTS AND FUTURE DIRECTIONS

Results from the 22q‐ENIGMA and ENIGMA‐CNV WGs confirm that multiple CNVs are associated with differences in brain morphology. This effort is providing information on genetically determined variation in brain development and its relation to neurodevelopmental, psychiatric, and neurological disorders. Current and future CNV studies will benefit both from even larger samples with more ethnicities represented, based on broad collaborations and standardized data collection across samples and in smaller, more flexible studies with deeper phenotyping.

### 7.1 Increasing sample sizes

There is great potential to include additional samples in ENIGMA WGs on CNVs. First, additional cohorts can easily be incorporated and additional measures such as the corpus callosum, cerebellum, brain stem, and ventricles—not yet targeted in ENIGMA‐CNV—can be added to the protocol. Second, independent research projects performing targeted recruitment and MRI brain scans on CNV carriers can easily join. Third, clinical scans of CNV carriers may be leveraged, provided the MRI quality is sufficient for accurate morphometry, and a number of appropriate genotyped controls from the same scanner site is provided: part of the standard evaluation for children with developmental delay or ID may include brain MRI, in particular for cases where additional clinical indications such as epilepsy, head circumference abnormalities or focal neurological signs are present (Mithyantha, Kneen, McCann, & Gladstone, 2017). Finally, such independent samples can be supplemented with an increasing amount of data from open data sets such as the ABCD study (Casey et al., 2018) and UK Biobank (Littlejohns et al., 2020).

There are notably analytical challenges and interpretational risks involved in large‐scale neuroimaging studies (Smith & Nichols, 2018). The initial ENIGMA studies on CNVs were able to capture effects across diverse, heterogenous study samples, but they might lack sensitivity to subtype‐specific effects that may be better captured by smaller well‐controlled studies focusing on targeted phenotyping. Likewise, the latter studies might be more suited to test new technology or methods. Nevertheless, a considerable strength lies in the ability of big studies to discover if effects generalize across samples collected all over the world. In other words, large‐scale studies and well‐designed smaller scale neuroimaging studies on CNVs offer useful complementary approaches.

### 7.2 Expanding the generalizability of data

#### 7.2.1 Clinical versus nonclinical effects and ancestry considerations

An increase in cross‐diagnostic data will allow a deeper insight into what distinguishes a CNV carrier with a severe phenotype (e.g., clinical diagnosis) from a well‐functioning carrier (typically with no clinical diagnosis). So far, effects of CNVs on the brain seem to be found in clinical and nonclinical carriers alike. With larger, more diverse samples that cover the entire phenotypic heterogeneity of all carriers, we may be able to deduce if a less severe clinical outcome is associated with a more moderate effect on the brain structural fingerprint.

Likewise, distinctions may be found for groups of different ancestry. So far, there is no evidence that CNVs have different effects across different ancestries but data are sparse, as most studies to date are based on white European samples; there is an urgent need to include individuals from diverse ancestries, to provide a broader and more inclusive view on this topic. Such cohorts are already being collected through several efforts, including initiatives on SCZ (Gulsuner et al., 2020) and neurodevelopment (de Menil et al., 2019).

#### 7.2.2 Lifespan trajectories

Current investigations have focused on the overall impact of CNVs on the brain, mostly disregarding a potential dynamic or interactive effect of age on brain maturation. Gross structural brain alterations are likely present from an early stage of development—as exemplified by the macrocephaly observed in utero in a 1q21.1 distal duplication carrier (Verhagen et al., 2015). However, detailed knowledge on the development of brain structure in CNV carriers over the lifespan is lacking. Recently, the first study to address this—targeting the 16p11.2 proximal CNV—suggested that differences in brain structure in deletion and duplication carriers in comparison to noncarriers remain stable throughout childhood, adolescence and at least until around 23 years of age (Cardenas‐de‐la‐Parra et al., 2019). Other evidence suggests that there may be differences in the profiles of neurodegeneration at older ages when carrying a CNV (Gentile, La Cognata, & Cavallaro, 2020) highlighting the need to study individuals across the lifespan.

### 7.3 Expanding phenotypic information

#### 7.3.1 Expanding to brain connectivity: Inclusion of DTI and resting state MRI

Accumulating evidence converges on brain dysconnectivity as a trans‐diagnostic phenotype in mental illness, based on aberrations in the “wiring” of the brain in individuals suffering from mental illness. To date, the 22q‐ENIGMA WG has targeted brain measures more broadly—including diffusion‐weighted MRI and surface‐based shape analysis—whereas the ENIGMA‐CNV WG has focused on ROI‐based measures of brain structure. Workflows for diffusion MRI analyses have already been developed and tested in various ENIGMA WGs (Jahanshad et al., 2013; Kochunov et al., 2016) including the 22q‐ENIGMA WG (Villalon‐Reina et al., 2019). Likewise, a resting‐state functional MRI processing pipeline was proposed recently for use in ENIGMA meta‐analyses (Adhikari et al., 2018). Future ENIGMA‐studies aimed at large scale investigations of structural and functional brain metrics aim to directly test the proposal of a “common symptom, common circuit” model of psychopathology in which genetic, epigenetic, and environmental risk factors affect connectivity in one or several neural circuits, producing cognitive, and emotional disturbances (Buckholtz & Meyer‐Lindenberg, 2012). So far, only a few studies have tested this concept in CNVs—either by doing analysis across pathogenic CNV carriers (Drakesmith et al., 2019), through analysis of severely affected individuals (22q11DS; Villalon‐Reina et al., 2019; Moreau et al., 2020), the 16p11.2 proximal (Chang et al., 2016; Moreau et al., 2020), or the most abundant low impact recurrent CNV, 15q11.2 (Silva et al., 2018). A combined effort within this arena would be likely to move the field forward.

#### 7.3.2 Structural covariation and spatial gene expression

The primary ENIGMA‐CNV and 22q‐ENIGMA imaging studies have provided a better understanding of the spatial distribution of CNV‐related brain alterations across the brain (localized to lobes, gyri, sulci, etc.). Methods such as structural covariance may help to better understand CNV‐related disruptions in the developmental coordination between maturing brain regions (Alexander‐Bloch, Raznahan, Bullmore, & Giedd, 2013). Techniques such as “virtual histology,” which relate group differences in MRI‐derived cortical measures (e.g., 22q11.2 carrier vs. healthy control) to gradients in cell‐specific gene expression from the Allen Human Brain Atlas may provide a step toward bridging the gap between MRI‐brain alterations and underlying cell‐specific pathophysiology (Patel et al., 2018; Shin et al., 2017).

#### 7.3.3 Adding clinical, cognitive, and behavioral data

The strength of the 22q‐ENIGMA and ENIGMA‐CNV WGs is combining large‐scale data on both CNV calls and imaging data. Adding deeper phenotyping information such as cognitive, mental, and behavioral data would be highly beneficial. The challenge with incorporating such data from independent studies is that the standardization of phenotypic information across the independently collected cohorts is lacking; other ENIGMA WGs have begun to deal with this challenge by harmonizing cognitive endpoints (Tate et al., 2021). Through organization and standardization of samples, we have the potential to deepen our knowledge regarding the relationships between CNV carriers, imaging measures and other phenotypes.

Publicly available data sets, such as the UK Biobank, already allow for large‐scale analysis of cross‐phenotypic traits—such as brain‐cognition mediation by combining cognition and brain imaging data. Given the continued recruitment of individuals for MRI studies, and the future availability of other large‐scale harmonized brain imaging data sets such as in the ABCD study (Casey et al., 2018), the potential of this type of analysis is continuously expanding. Future studies aim to move beyond case–control comparisons to better understand the underlying brain structure and functional mechanisms related to behavioral phenotype heterogeneity in CNV carriers (Marquand, Rezek, Buitelaar, & Beckmann, 2016). These types of analysis, taking advantage of “big data” samples, call for robust data‐driven approaches that do not simply maximize prediction accuracy but improve our interpretation and understanding of underlying mechanisms (Bzdok, Nichols, & Smith, 2019).

#### 7.3.4 Identifying causal mechanisms—Mechanistic follow‐up studies

Candidate genes within the CNV regions can be linked to neuronal differentiation, axon outgrowth and dendritic spine formation, neurotransmitter and immune signaling, and other processes important for brain development. Moving forward, interest will no doubt focus on identifying and characterizing these driver genes causing the phenotypes or factors modifying the phenotypes. Many approaches can aid in this effort.

Studies of variable CNV breakpoints can narrow down potential driver genes. This is exemplified by the recent dismissal of *HYDIN2* as a driver gene for the head circumference phenotype in 1q21.1 distal CNV: Several atypical 1q21.1 distal deletion and duplication carriers with normal copy number variation of *HYDIN2*, but still exhibiting the microcephaly or macrocephaly phenotype, were identified (Dougherty et al., 2017). A recent exome sequencing study of people with autism identified *BCL11A* as a potential driver gene for autism in the 2p15‐p16.1 CNV (Satterstrom et al., 2020). Likewise, gene expression studies have helped to narrow down the gene behind the SNP association for brain structure. Another option is expression of individual genes in model organisms such as mouse (Dominguez‐Iturza et al., 2019; Nielsen et al., 2017) or zebrafish (Loviglio et al., 2017). Such approaches may identify driver genes for brain phenotypes associated with CNVs, pinpointing the biological mechanisms involved.

Finally, future studies should try to disentangle the role of common genetic variants in moderating the phenotype caused by CNVs, through common and rare variant interplay analysis as done for instance in SCZ (Bergen et al., 2018; Tansey et al., 2016) and ADHD (Martin et al., 2015).

### 7.4 Secondary proposals

Several secondary projects are ongoing in the 22q‐ENIGMA and ENIGMA‐CNV WGs. In the 22q‐ENIGMA WG, a secondary project led by Fidel Vila‐Rodriguez (The University of British Columbia) is now investigating the structural covariance of gray matter volume using source‐based morphometry, and another study led by Jennifer Forsyth at UCLA is investigating specific 22q11.2 genes driving cortical surface area and thickness alterations.. In addition to recurrent CNVs, numerous single, nonrecurrent CNVs disrupting one or more genes may have a large impact on the brain and behavior but determining the impact and clinical interpretation of these single CNVs is even more challenging (Nowakowska, 2017). In ENIGMA‐CNV, “The effect of very rare CNVs on brain structure and function,” headed by the group of Sébastien Jacquemont at University of Montreal, investigates very rare CNVs by analyzing all CNVs in the genome—even single hit CNVs—and their effect on brain structure. Another secondary project in the ENIGMA‐CNV WG, headed by David Linden (Cardiff University/Maastricht University), addresses how brain changes across different pathogenic CNVs correlate with penetrance scores for SCZ and developmental delay, aiming to find common brain phenotypes that are most related to risk for disorders.

## 8 CONCLUSION

CNV analysis offers a genetics‐first approach to studying neurodevelopmental, neurological and neuropsychiatric disorders as well as related traits. Using convergent evidence from neuropsychological testing, structural and functional neuroimaging, the 22q‐ENIGMA and ENIGMA‐CNV WGs have mapped the effects of CNVs in some of the largest neuroimaging data sets ever analyzed, revealing consistent brain signatures of CNVs, overlap with idiopathic disorders and, in the case of 22q11DS, its relation to psychotic conditions. Although these studies have revealed consensus associations between varying CNVs and brain structure, much remains to be explored. Data sharing and international collaborations are essential for CNV studies, and the large‐scale efforts conducted by the 22q‐ENIGMA WG, the ENIGMA‐CNV WG and Psychiatric Genomics Consortium serve as an example of how team science can tackle some of these core challenges facing CNV research.

### 8.1 Join the CNV efforts of ENIGMA

The ENIGMA‐CNV and 22q‐ENIGMA WGs continue to recruit new participating members, and the infrastructure allows for new groups to be efficiently incorporated into ongoing and future analyses. Groups interested in joining or contributing data are encouraged to contact the WG Chairs. This needs to be a community effort—so we encourage everyone to join the effort. Because every CNV carrier counts.

## 9 CONTACT DETAILS

Those interested are invited to contact the coordinators:

ENIGMA‐CNV WG: Ida Elken Sønderby & Ole A. Andreassen. Webpage: http://enigma.ini.usc.edu/ongoing/enigma-cnv/.

22q‐ENIGMA WG: Carrie E. Bearden and Christopher R. K. Ching. Webpage: http://enigma.ini.usc.edu/ongoing/enigma-22q-working-group/

## DISCLOSURE OF INTERESTS

O. A. A. is a Speaker's honorarium at Lundbeck, Consultant at HealthLytix. K. M. A. has received investigator‐initiated research funds from Shire and has participated in an advisory panel for Arbor Pharmaceuticals. C. A. has been a consultant to or has received honoraria or grants from Acadia, Angelini, Gedeon Richter, Janssen Cilag, Lundbeck, Minerva, Otsuka, Roche, Sage, Servier, Shire, Schering Plough, Sumitomo Dainippon Pharma, Sunovion, and Takeda. P. M. T. and C. R. K. C. have received partial research support from Biogen, Inc. (Boston) for work unrelated to the topic of this manuscript. D. P. H. is a full‐time employee of Genentech, Inc. A. M. D. is a Founder of and holds equity in CorTechs Labs, Inc, and serves on its Scientific Advisory Board. H. S., M. O. U., G. B. W., K. S. are employees of deCODE genetics/Amgen. H. J. G. has received travel grants and speakers honoraria from Fresenius Medical Care, Neuraxpharm, Servier, and Janssen Cilag as well as research funding from Fresenius Medical Care. JH has received speakers honoraria from Lilly, Biocodex, HB‐Pharma, Medice, Takeda, and Shire. D. J. S. has received research grants and/or consultancy honoraria from Lundbeck and Servier. F. V.‐R. receives research support from CIHR, Brain Canada, Michael Smith Foundation for Health Research, Vancouver Coastal Health Research Institute, and in‐kind equipment support for this investigator‐initiated trial from MagVenture. F. V.‐R. has also participated in an advisory board for Janssen.

## Data Availability

Data sharing is not applicable to this article as no new data were created or analyzed in this study.

## ENIGMA‐CNV Additional Members

Manon Bernard, Bloorview Research Institute, Holland Bloorview Kids Rehabilitation Hospital, Toronto, Ontario, Canada.

Nicholas B. Blackburn, Department of Human Genetics, School of Medicine, University of Texas Rio Grande Valley, Brownsville, Texas, USA; South Texas Diabetes and Obesity Institute, School of Medicine, University of Texas Rio Grande Valley, Brownsville, Texas, USA.

Rune Bøen, Department of Medical Genetics, Oslo University Hospital, Oslo, Norway; Norwegian Centre for Mental Disorders Research (NORMENT), Division of Mental Health and Addiction, Oslo University Hospital and University of Oslo, Oslo, Norway.

Eco de Geus, Department of Biological Psychology, Vrije Universiteit Amsterdam, Amsterdam, The Netherlands; Amsterdam Public Health (APH) Research Institute, Amsterdam UMC, Amsterdam, The Netherlands.

Sonja M. C. de Zwarte, Department of Psychiatry, UMC Utrecht Brain Center, University Medical Center Utrecht, Utrecht University, Utrecht, The Netherlands.

Marta Di Forti, Institute of Psychiatry, Psychology & Neuroscience, King's College London, London, UK.

Oleksandr Frei, Norwegian Centre for Mental Disorders Research (NORMENT), Division of Mental Health; Addiction, Oslo University Hospital and University of Oslo, Oslo, Norway and Center for Bioinformatics, Department of Informatics, University of Oslo, Oslo, Norway, Vincent Frouin, Université Paris‐Saclay, CEA, Neurospin, Gif‐sur‐Yvette, France.

Masaki Fukunaga, Division of Cerebral Integration, National Institute for Physiological Sciences, Okazaki, Japan.

Jayne Y. Hehir‐Kwa, Princess Máxima Center for Pediatric Oncology, Utrecht, The Netherlands.

Manon H. J. Hillegers, Department of Child and Adolescent Psychiatry/Psychology, Erasmus Medical Center, Rotterdam, The Netherlands.

Per Hoffmann, Institute of Human Genetics, University of Bonn, Bonn, Germany; Human Genomics Research Group, Department of Biomedicine, University of Basel, Basel, Switzerland.

Georg Homuth, Interfaculty Institute for Genetics and Functional Genomics, University Medicine Greifswald, Greifswald, Germany.

Neda Jahanshad, Imaging Genetics Center, Mark and Mary Stevens Neuroimaging and Informatics Institute, Keck School of Medicine, University of Southern California, Marina del Rey, CA, USA.

Sanne Koops, Department of Biomedical Sciences of Cells & Systems, Cognitive Neurosciences, University of Groningen, University Medical Center Groningen (UMCG), Groningen, The Netherlands.

Kuldeep Kumar, Sainte Justine Research Center, University of Montreal, Montreal, QC, Canada.

Masataka Kikuchi, Graduate School of Medicine, Osaka University, Osaka, Japan.

Stephanie Le Hellard, NORMENT, Department of Clinical Science, University of Bergen, Bergen, Norway; Dr. Einar Martens Research Group for Biological Psychiatry, Department of Medical Genetics, Haukeland University Hospital, Bergen, Norway.

Costin Leu, Genomic Medicine Institute, Lerner Research Institute, Cleveland Clinic, Cleveland, OH, USA; Department of Clinical and Experimental Epilepsy, UCL Queen Square Institute of Neurology, London, UK; Stanley Center for Psychiatric Research, Broad Institute of Harvard and M.I.T, Cambridge, MA, USA.

Robin M Murray, Institute of Psychiatry, Psychology & Neuroscience, King's College London, London, UK.

Terje Nærland, KG Jebsen Centre for Neurodevelopmental Disorders, University of Oslo, Oslo, Norway; NevSom, Department of Rare Disorders, Oslo University Hospital, Oslo, Norway.

Lars Nyberg, Integrative Medical Biology (IMB) Umeå University, Umeå, Sweden; Department of Psychiatry, Erasmus University Medical Center, Rotterdam, The Netherlands.

Roel A. Ophoff, Center for Neurobehavioral Genetics, University of California Los Angeles, Los Angeles, CA, USA; Department of Psychiatry, Erasmus University Medical Center, Rotterdam, The Netherlands.

G Bruce Pike, Radiology and Clinical Neurosciences, University of Calgary, Calgary, AB, Canada.

Sigrid B. Sando, Department of Neuromedicine and Movement Science, Faculty of Medicine and Health Sciences, Norwegian University of Science and Technology, Trondheim, Norway; Department of Neurology and Clinical Neurophysiology, University Hospital of Trondheim, Trondheim, Norway.

Jean Shin, The Hospital for Sick Children, Toronto, ON, Canada; Departments of Physiology and Nutritional Sciences, University of Toronto, Toronto, Ontario, Canada.

Elena Shumskaya, Donders Institute for Brain, Cognition, and Behavior, Nijmegen, The Netherlands.

Sanjay M. Sisodiya, Department of Clinical and Experimental Epilepsy, UCL Queen Square Institute of Neurology, London, UK; Chalfont Centre for Epilepsy, Chalfont‐St‐Peter, Bucks, UK.

Vidar M. Steen, NORMENT, Department of Clinical Science, University of Bergen, Bergen, Norway; Dr. Einar Martens Research Group for Biological Psychiatry, Department of Medical Genetics, Haukeland University Hospital, Bergen, Norway.

Alexander Teumer, Institute for Community Medicine, University Medicine Greifswald, Greifswald, Germany,

Anne Uhlmann, Department of Psychiatry and Mental Health, University of Cape Town, Cape Town, South Africa.

Margaret J. Wright, Queensland Brain Institute, The University of Queensland, Brisbane, QLD, Australia

## ENIGMA 22q11.2 Deletion Syndrome Additional Members

Kevin M. Antshel, Department of Psychology, Syracuse University, Syracuse, NY, USA.

Linda E. Campbell, University of Newcastle, Newcastle, NSW, Australia.

Nicolas A. Crossley, Department of Psychiatry, Pontificia Universidad Católica de Chile, Santiago, Chile.

T. Blaine Crowley, Division of Human Genetics and 22q and You Center, Children's Hospital of Philadelphia, Philadelphia, PA, USA.

Eileen Daly, Department of Forensic and Neurodevelopmental Sciences, and the Sackler Institute for Translational Neurodevelopmental Sciences, Institute of Psychiatry, Psychology and Neuroscience, King's College, London, UK.

Ania M. Fiksinski, Clinical Genetics Research Program, Centre for Addiction and Mental Health, Toronto, ON, Canada; Dalglish Family 22q Clinic for Adults with 22q11.2 Deletion Syndrome, Toronto General Hospital, University Health Network, Toronto, ON, Canada; Department of Psychiatry, UMC Utrecht Brain Center, University Medical Center Utrecht, Utrecht University, Utrecht, The Netherlands.

Jennifer K. Forsyth, Department of Psychiatry and Biobehavioral Sciences, University of California Los Angeles, Los Angeles, CA, USA.

Wanda Fremont, Department of Psychiatry and Behavioral Sciences, SUNY Upstate Medical University, Syracuse, NY, USA.

Naomi J. Goodrich‐Hunsaker, MIND Institute and Department of Psychiatry and Behavioral Sciences, University of California Davis, Davis, CA, USA; Department of Neurology, University of Utah, Salt Lake City, UT, USA.

Maria Gudbrandsen, Department of Forensic and Neurodevelopmental Sciences, and the Sackler Institute for Translational Neurodevelopmental Sciences, Institute of Psychiatry, Psychology and Neuroscience, King's College, London, UK.

Rachel K. Jonas, Semel Institute for Neuroscience and Human Behavior, Departments of Psychiatry and Biobehavioral Sciences and Psychology, University of California Los Angeles, Los Angeles, CA, USA.

Wendy R. Kates, Department of Psychiatry and Behavioral Sciences, State University of New York at Upstate Medical University, Syracuse, NY, USA.

Amy Lin, Semel Institute for Neuroscience and Human Behavior, University of California Los Angeles, Los Angeles, CA, USA.

Kathryn L. McCabe, Department of Psychiatry and Behavioral Sciences, State University of New York at Upstate Medical University, Syracuse, NY, USA; Department of Psychology, University of Newcastle, Newcastle, NSW, Australia.

Hayley Moss, MRC Centre for Neuropsychiatric Genetics and Genomics, Division of Psychological Medicine and Clinical Neurosciences, Cardiff University, Cardiff, UK.

Declan G. Murphy, Department of Forensic and Neurodevelopmental Sciences, and the Sackler Institute for Translational Neurodevelopmental Sciences, Institute of Psychiatry, Psychology and Neuroscience, King's College, London, UK; Behavioural Genetics Clinic, Adult Autism Service, Behavioural and Developmental Psychiatry Clinical Academic Group, South London and Maudsley NHS Foundation Trust, London, UK.

Kieran C. Murphy, Department of Psychiatry, Royal College of Surgeons in Ireland, Dublin, Ireland.

Michael J. Owen, MRC Centre for Neuropsychiatric Genetics and Genomics, Division of Psychological Medicine and Clinical Neurosciences, Cardiff University, Cardiff, UK.

Kosha Ruparel, Department of Psychiatry, Perelman School of Medicine at the University of Pennsylvania, Philadelphia, PA, USA.

Tony. J. Simon, MIND Institute and Department of Psychiatry and Behavioral Sciences, University of California Davis, Davis, CA, USA.

Therese van Amelsvoort, Department of Psychiatry and Neuropsychology, Maastricht University, Maastricht, The Netherlands.

Jacob A. S. Vorstman, Department of Psychiatry, The Hospital for Sick Children, Toronto, ON, Canada.
